# Neutrophil-based delivery platforms: from natural mechanisms to engineered therapeutics

**DOI:** 10.7150/thno.117363

**Published:** 2026-01-01

**Authors:** Yinggang Li, Xue Zhang, Yunkun Li, Ling Sun, Na Hu, Su Lui, Zhenyu Duan, Qiyong Gong, Kui Luo

**Affiliations:** 1 Department of Radiology, Huaxi MR Research Center (HMRRC), Institution of Radiology and Medical Imaging, Rehabilitation Therapy, Institute of Breast Health Medicine, Frontiers Science Center for Disease-Related Molecular Network, State Key Laboratory of Biotherapy, West China Hospital, Sichuan University, Chengdu 610041, China.; 2 Psychoradiology Key Laboratory of Sichuan Province, Key Laboratory of Transplant Engineering and Immunology, NHC, and Research Unit of Psychoradiology, Chinese Academy of Medical Sciences, Chengdu 610041, China.; 3 Xiamen Key Lab of Psychoradiology and Neuromodulation, Department of Radiology, West China Xiamen Hospital of Sichuan University, Xiamen 361021, China.

**Keywords:** neutrophil, nanomedicine, targeted drug delivery, biomimetic, immunometabolism

## Abstract

Neutrophils are one of the key components of the innate immune system, and they play essential roles in various physiological processes, including phagocytosis, chemotaxis, immune sensing, and transmigration across the vascular endothelium. The synergy of neutrophil biology with nanomaterial science has led to the development of innovative neutrophil-based drug delivery systems (NDDSs) or neutrophil-derived biomimetic delivery systems. In this review, we elucidate the mechanisms underlying neutrophil-mediated targeting strategies. By utilizing inherent properties of neutrophils, targeted delivery to specific disease sites through NDDSs can be achieved. We survey various approaches for constructing NDDSs via live cell delivery strategies involving cell loading, *in vivo* capture, surface modification, and gene editing, as well as neutrophil-mimicking approaches based on neutrophil membranes, exosomes, and neutrophil-like cells. Manipulation of drug loading and release from NDDSs and functionalization of neutrophils allow for precise regulation and intervention of disease procession. In addition, we propose emerging approaches for novel NDDSs from an immunometabolic perspective. Finally, we address challenges and opportunities for advancing NDDSs into clinical practice.

## 1. Introduction

The emergence of drug delivery systems has enabled controlled drug distribution and release, reduced toxicity and improved therapeutic efficacy 1-5. Traditional drug delivery systems have been developed, including liposomes, polymeric nanoparticles (NPs), dendrimers, albumin NPs, and other carriers 6-12. With the integration of biology and nanomedicine, a variety of biologically derived drug delivery systems, including those derived from bacteria, exosomes, live cells, and viruses, have emerged 6, 13. These natural systems offer unique properties that synthetic materials cannot provide, positioning them as promising drug delivery platforms. Among these natural systems, mammalian cell types, including nature killer (NK) cells, macrophages, T cells, red blood cells, and cancer cells have been explored as drug delivery carriers 14-18. Neutrophils, the most abundant white blood cells, play a crucial role in immune defense and disease progression 19, 20. Their rapid response to infection, site-specific chemotaxis, and active phagocytosis allow them to efficiently deliver therapeutic agents to targeted disease sites 21. Leveraging these unique physiological functions of neutrophils, NDDSs can precisely overcome biological barriers and improve treatment efficacy of the incorporated drugs in NDDSs.

Several modifications have been developed to enhance NDDSs, further showcasing the potential of these systems in therapeutic applications. For example, NDDSs often rely on their phagocytic ability to engulf drugs or NPs, creating a “Trojan horse” effect 22. In addition, activated neutrophils are able to cross the blood-brain barrier (BBB) 23, and aged neutrophils can home to the bone marrow 24 and accumulate at surgical sites 25. By employing neutrophils in different states, targeting of specific sites could be achieved by NDDSs. Therefore, effective NDDSs could be developed by harnessing neutrophil kinetics and their behavior in specific physiological states. Emerging technologies, such as drug “backpacks”, gene editing, and immune training, can endow NDDSs with advanced functionalities. For example, chimeric antigen receptor (CAR) neutrophils can be engineered for targeted cytolysis of specific cells, while release of specific cytokines from drug backpacks can modulate neutrophil phenotypes for anti-tumor activity. Neutrophil-mimicking drug delivery systems, for instance, with a coating of neutrophil membranes, can preserve the characteristics of neutrophils, such as tissue targeting and improved immune evasion 26-28. So far, NDDSs and neutrophil-derived biomimetic delivery systems have been developed to treat cancer, cardiovascular diseases, inflammation, and bacterial infections and these delivery systems have been demonstrated with great promise in animal models.

**Figure 1:**
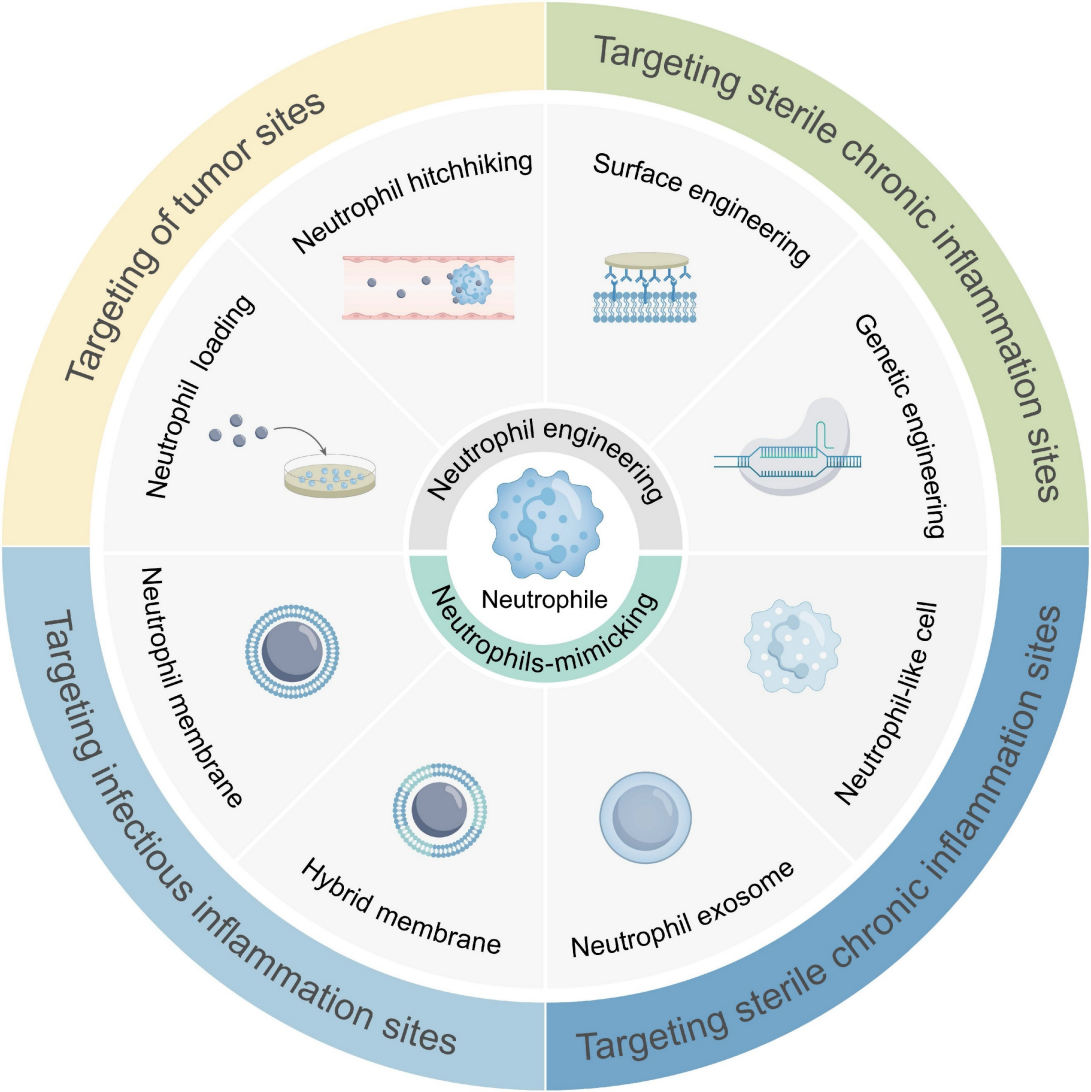
** Schematic illustration of targeted therapy at lesion sites via engineered neutrophils-mediated or neutrophil-mimicking drug delivery systems.** Eight strategies have been developed for engineered neutrophil-mediated/mimetic drug delivery systems: *in vitro* neutrophil drug loading; *in vivo* neutrophil hitchhiking; genetic engineering of neutrophils; surface-engineering of neutrophils; neutrophil membrane-coated systems; hybrid membrane systems; neutrophil exosome systems; neutrophil-like cell systems. These systems have been demonstrated to enable precise delivery to inflammatory diseases, tumors, CNS pathologies, orthopedic disorders, and post-surgical sites.

In this review, the roles of neutrophils in the human body were briefly introduced, and the mechanisms of targeting different disease sites were elaborated. We then introduced various strategies for drug loading into neutrophils, including *ex vivo* loading, *in vivo* hijacking, genetic modification, drug backpacks, and immune training. Additionally, neutrophil-mimicking approaches, such as neutrophil membranes, hybrid neutrophil membranes, neutrophil-derived exosomes, and neutrophil-like cells, were discussed. From an immunometabolic perspective, we proposed a new direction for designing innovative NDDSs. Finally, we presented opportunities and challenges in developing NDDSs. Discovery of neutrophil biological/physiological functions and unveiling of their targeting mechanisms will pave the way for the development of effective and precise drug delivery systems (**Figure 1**).

## 2. Physiological Basis of Neutrophils for Drug Delivery

**Figure 2:**
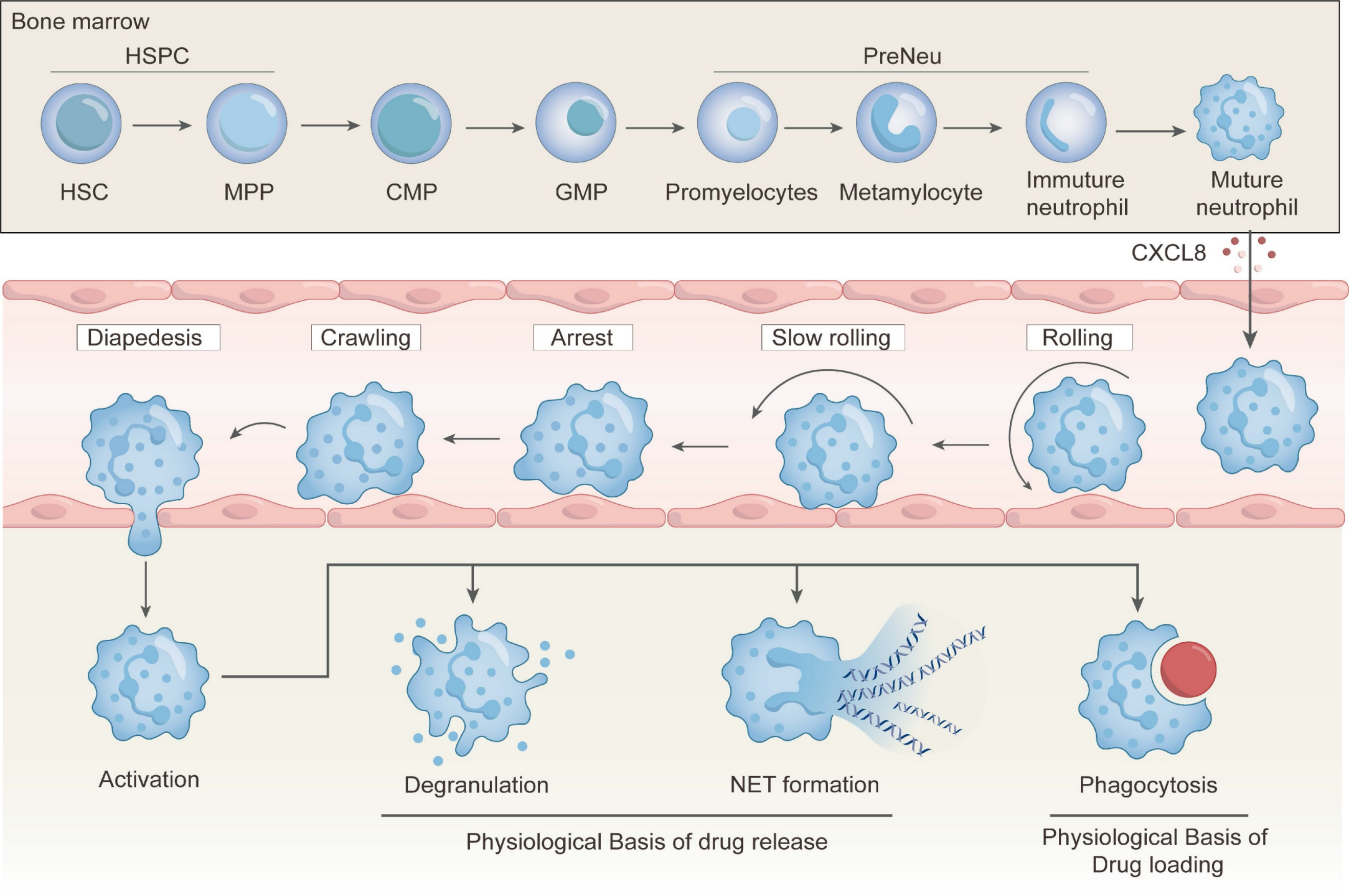
**Physiological basis of neutrophils as a drug delivery system.** Neutrophils originate and mature in the bone marrow before entering systemic circulation. Upon encountering inflammatory cues, they undergo a well-orchestrated sequence of migration steps—rolling, slow rolling, arrest, crawling, and diapedesis—to infiltrate affected tissues. Once activated, neutrophils can release therapeutics via detachment and NETs formation. Notably, phagocytosis during this process provides a physiological basis for drug loading and release.

Neutrophils possess distinct biological properties that can be particularly harnessed for drug delivery to inflammatory and tumor sites. As first-line responders to acute inflammation, they exhibit rapid and robust chemotactic migration, and they can efficiently home to sites of infection or tumors. Additionally, neutrophils are the most abundant leukocytes in human blood in which there are 50-70% of circulating white cells. They are produced continuously in the bone marrow at a rate of up to 2 × 10¹¹ cells per day 19, 29, 30. Unlike macrophages that require time-consuming in vitro differentiation, neutrophils can be readily isolated from peripheral blood, providing practical advantages for rapid and scalable drug delivery application. Employment of neutrophils as a drug delivery component could be a promising strategy for achieving targeted drug delivery because they can home to pathological sites, internalize nanoparticles (NPs), and release cargos via degranulation or neutrophil extracellular traps (NETs) formation (**Figure 2**). In this section, biological mechanisms underlying the use of neutrophils for drug delivery systems are elaborated, and these mechanisms lay the foundation for rational design and clinical translation of NDDSs.

### 2.1 Physiological basis of drug loading into neutrophils

Phagocytosis is widely regarded as the primary pathway for NPs uptake by neutrophils 31, 32. This process relies on surface receptors such as Toll-like receptors (TLRs), Fc gamma receptors (FcγRs), and integrin αvβ1. It has been shown that TLRs capture components such as Lipopolysaccharide (LPS) and CpG (Cytosine-Guanine dinucleotide), FcγRs mediate immune complex phagocytosis, and integrin αvβ1 promotes NPs uptake through specific ligand interactions 33. These receptors recognize pathogen-associated molecular patterns (PAMPs) and facilitate effective engulfment or internalization of pathogens or NPs-bound pathogens 34. Therefore, neutrophils could be served as effective drug carriers by loading drugs through phagocytosis. However, phagocytosis involves the engulfment of pathogens into membrane-bound phagosomes, where neutrophils exert intracellular antimicrobial activity through production of NADPH oxidase-dependent reactive oxygen species (ROS) and activation of lysosomal enzymes such as lysozyme, lactoferrin, cathepsins, and defensins. Simultaneously, neutrophils can eliminate pathogens extracellularly via the release of antimicrobial proteins 35. Therefore, when neutrophils are employed to carry NPs, the interaction between NPs and these enzymes should be examined to prevent premature release of NPs from neutrophils.

In addition, the efficiency of internalizing nano-sized NPs by neutrophils via phagocytosis varies from different studies, and there may be alternative internalization pathways for neutrophils. NPs can enter neutrophils via non-phagocytic endocytic pathways, including clathrin-mediated endocytosis, caveolin-mediated endocytosis, ADP-ribosylation factor 6-dependent endocytosis, and micropinocytosis 36-38. These endocytic mechanisms are often dependent on the size and surface characteristics of NPs. For instance, clathrin-mediated endocytosis predominantly regulates the uptake of NPs in the size range of 100-150 nm, while large particles ranging from 250 nm to 5 µm are often internalized via macropinocytosis 38-40. Furthermore, the activation state of neutrophils has a significant impact on their uptake capacity for NPs. It has been demonstrated that inflammatory stimuli, such as TNF-α or LPS, markedly enhance internalization of NPs. For instance, in inflammatory tissues, approximately 30% of infiltrating neutrophils internalize albumin NPs within 20 h post-injection, whereas resting or circulating neutrophils barely internalize these NPs 38, 39. This suggests that activated neutrophils can facilitate on-site internalization of NPs through phagocytosis and other endocytosis pathways, thereby promoting accumulation of NPs at disease sites. In summary, NPs enter neutrophils via distinct pathways depending on their physicochemical properties, and the neutrophil uptake capacity is dependent upon different physiological conditions.

### 2.2 Neutrophil development and migration mechanisms

Neutrophil development initiates from hematopoietic stem cells (HSCs) in the bone marrow, which possess self-renewal and differentiation capacities. Activated HSCs differentiate into multipotent progenitors (MPPs), collectively forming the pool of hematopoietic stem and progenitor cells (HSPCs). MPPs can differentiate into common myeloid progenitors (CMPs), which further differentiate into granulocyte-monocyte progenitors (GMPs) 41, 42. GMPs create a proliferative pool of neutrophil-committed preneutrophils, which subsequently differentiate into non-proliferative immature neutrophils and ultimately mature neutrophils 43. These mature neutrophils are released from the bone marrow into the circulation system. These differentiation steps are characterized with changes in the nuclear morphology, progressing from round (promyelocytes) to kidney-shaped (metamyelocytes), band-shaped (immature neutrophils), and segmented (mature neutrophils). Mature neutrophils are retained in the bone marrow through the interaction between stromal cell-derived CXCL12 and C-X-C chemokine receptor type 4 (CXCR4) 44, 45. Their release into the circulation system is regulated by granulocyte colony-stimulating factor (G-CSF). G-CSF induces degradation of CXCL12 by proteases (e.g., elastase), downregulation of CXCR4, and upregulation of CXCR2. These coordinated events promote neutrophil detachment from the marrow stroma and transmigration across the sinusoidal endothelium. This process can be accelerated by an elevated level of G-CSF during infection or inflammation 46.

The migration and chemotaxis mechanisms of neutrophils are the cornerstone of the NDDSs. In response to inflammatory signals, neutrophils exit the bloodstream and infiltrate interstitial tissues via a sequential cascade: capture, rolling, slow rolling, adhesion, crawling, and transendothelial migration (**Figure 2**) 47-49. Initially, inactive neutrophils are rapidly activated upon encountering inflammatory cytokines, triggering the adhesion cascade. Inflammatory signals upregulate endothelial selectins, which mediate initial low-affinity tethering with neutrophil receptors. The tethering triggers intracellular signaling of neutrophils. Chemokine-driven integrin activation then promotes firm adhesion and cytoskeletal remodeling. Finally, neutrophils polarize and crawl laterally over the endothelium to arrive at an optimal site for transmigration via LFA-1 (αLβ2), Mac-1 (αMβ2) and VLA-4 (α4β1) binding to intercellular adhesion molecule-1 (ICAM-1), intercellular adhesion molecule-2 (ICAM-2) and vascular cell adhesion molecule-1 (VCAM-1), respectively 50. After crawling and polarization, actin filaments in neutrophils are reorganized to form protrusions. These protrusions are essential to sense and respond to the chemotactic gradient, thus they can traverse various layers, including the endothelial surface, pericyte layers, and the basal membrane, to deeper penetrate tissues 51.

### 2.3 Drug release mediated by neutrophil degranulation and NETs

After reaching the tissue interstitium, drugs are released from NDDSs to exert their therapeutic action on targeted cells. It is noteworthy that specific targeting of tumors by neutrophils allows precise drug release at the tumor site. It has been widely accepted that there are two primary mechanisms of releasing drugs from the NDDSs 43-45. The first mechanism of drug release is through degranulation. Degranulation occurs in response to extracellular infection. When granules fuse with the cell membrane, antimicrobial proteins are released into the phagosome or the extracellular space, thereby targeting intracellular or extracellular pathogens, respectively 52. This process can be harnessed for drug release from NPs in neutrophils. When granules fuse with the cell membrane, encapsulated drugs could be released into the extracellular environment 53. However, it is questionable for the mechanism of drug release via degranulation, and more mechanistic studies should be conducted for a deep understanding of drug release through degranulation.

The second mechanism involves the formation of NETs. Highly activated neutrophils can eliminate extracellular microbes by releasing NETs, which are composed of DNA strands along with histones, antimicrobial proteins (such as lactoferrin and cathepsins), and enzymes (such as MPO and neutrophil elastase) derived from granules 54. NETs can trap pathogens, preventing their spread and promoting subsequent phagocytosis. Furthermore, NETs could directly kill pathogens through the antimicrobial actions of histones and proteases 54-56. Neutrophil-based drug delivery strategies often leverage the NETs formation process for controlled drug release. It has been shown that NETs can capture anticancer drugs such as doxorubicin (DOX) and paclitaxel (PTX), as well as highly active DNA-binding agents like mitomycin C or platinum-based drugs. However, NETs have been demonstrated to encapsulate tumor cells or adhere to them, which promote tumor dissemination and metastasis. NETs can also activate dormant tumor cells, facilitating tumor recurrence 57. Consequently, the drug delivery systems based on the NET release process should be carefully designed to avoid potential side effects associated with the NET release process. When neutrophils deliver drugs to the tumor site, the released drugs may not effectively penetrate deep tumor tissues. Thus, poor intratumoral distribution of these drugs remains a key challenge. Therefore, the mechanism underlying the release of loaded drugs in neutrophils should be systematically examined. The development of NDDSs is still in the early stage and many challenges remain to be addressed 58.

To achieve precise drug release in NDDSs, it is essential to maintain drug stability within neutrophils during circulation and ensure responsive release at pathological sites. This can be accomplished by incorporating stimuli-responsive chemical linkages that be responsive to specific pathological microenvironment cues, such as pH, redox potential, or enzyme activity 59-66. Additionally, camouflaging nanoparticles with *E. coli* membranes has been shown to enhance drug retention within neutrophils, preventing premature leakage 65, 67. External stimuli, such as near-infrared irradiation, can also be employed to induce controlled drug release by disrupting neutrophil structures at the target site 68.

## 3. Mechanisms of Targeting Pathological Regions by Neutrophils

**Figure 3:**
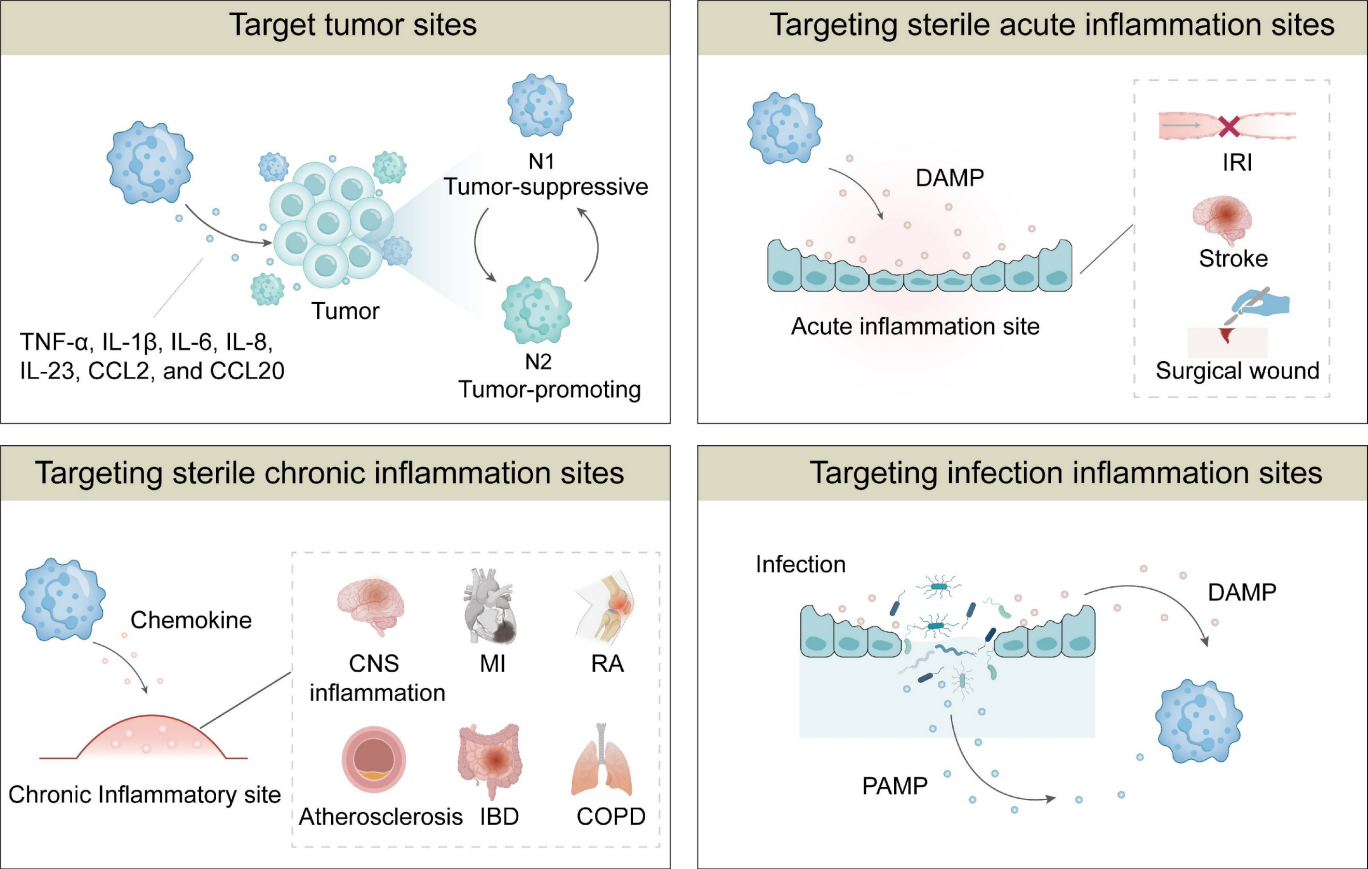
**Mechanisms of targeting distinct pathological sites by neutrophils.** Neutrophils exhibit intrinsic targetability toward various pathological sites via different mechanisms, including targeting tumor sites, sterile acute inflammation sites, sterile chronic inflammation sites, and infection inflammation sites.

Neutrophils possess inherent properties that render them effective drug carriers, which also allow controlled drug release at inflammatory sites. Their unique properties include targeting tumors, sterile acute inflammation, sterile chronic inflammation and infection inflammation 23-25, 69. Neutrophils are capable of rapidly travelling to pathological sites within the body due to their involvement in various diseases. More importantly, they exhibit distinct targetability towards specific organs or tissues under different pathological conditions. Consequently, NDDSs could enable localized delivery of therapeutics to target sites. The targeting mechanisms of neutrophils to different diseases are summarized in **Figure 3**.

### 3.1 Targeting tumors

The inflammatory nature of the microenvironment (TME) plays a critical role in modulating neutrophil recruitment, activation, and polarization, and it influences both tumor progression and therapeutic interventions 70. Tumors often exhibit elevated levels of pro-inflammatory cytokines and chemokines, including TNF-α, IL-1β, IL-6, IL-8, IL-23, CCL2, and CCL20, which create a favorable environment to promote neutrophil chemotaxis and infiltration from the bloodstream into tumor sites. Neutrophils express a variety of adhesion molecules, including L-selectin, lymphocyte function-associated antigen 1 (LFA-1), β1 integrin, CXCR4, macrophage-1 antigen (Mac-1), platelet endothelial cell adhesion molecule-1 (PECAM-1), and P-selectin glycoprotein ligand-1 (PSGL-1) 71-73. These adhesion receptors mediate neutrophils tethering, firm adhesion and transmigration via high-affinity interactions with endothelial cell surface ligands, which facilitate the migration of NDDSs from the blood stream into tumor vasculature.

Upon infiltration, neutrophils undergo phenotypic polarization, giving rise to two distinct subtypes: tumor-suppressive N1 neutrophils and tumor-promoting N2 neutrophils. N1 neutrophils exhibit a pro-inflammatory, anti-tumor phenotype characterized by enhanced cytotoxicity, production of ROS and reactive nitrogen species (RNS), and activation of CD8^+^ T cells 74. Additionally, N1 neutrophils facilitate the recruitment of M1 macrophages, further amplifying the immune response against tumors. By contrast, N2 neutrophils display an immunosuppressive phenotype, supporting tumor angiogenesis, invasion, and metastasis 75, 76. These neutrophils suppress CD8^+^ T cell and NK cell activity and promote the recruitment of M2 macrophages and regulatory T cells, thereby facilitating immune evasion and tumor progression 77. While neutrophils have been considered to play a role in tumor promotion and poor prognosis, accumulating evidence suggests that they can also exert anti-tumor effects under specific conditions. In early-stage tumors and pre-metastatic niches, neutrophils have been shown to suppress tumor growth and enhance anti-tumor immunity by stimulating NK cells and other T cell subsets 78. The paradoxical roles of neutrophils in cancer may stem from the dynamics of the TME, which dictates their activation, differentiation, and functional plasticity. To harness the therapeutic potential of neutrophils, their phenotype should be modulated toward an anti-tumor state.

### 3.2 Targeting sterile acute inflammation

Acute inflammation is a rapid, short-lived host response to tissue injury or infection, characterized by vasodilation, increased vascular permeability, and leukocyte extravasation. It initiates a complex cascade of signaling events to establish chemotactic gradients that orchestrate rapid recruitment of neutrophils to damaged tissue sites. Acute inflammation, commonly associated with clinical scenarios such as stroke, surgical trauma, and particularly organ transplantation, represents a prototypical model of acute sterile inflammation characterized by robust neutrophil infiltration. NDDSs have been reported to play a role in modulating such inflammatory responses 79-82. By delineating cellular and molecular mechanisms underlying different forms of acute inflammation, one can develop targeted strategies to modulate neutrophil recruitment and activation.

Ischemia-reperfusion injury (IRI) is a typical example of acute sterile inflammation and it is a major contributor to early graft dysfunction after organ transplantation. Upon reperfusion, recipient-derived neutrophils rapidly mobilize and accumulate in the graft vasculature, as early as 2-3 hours post-transplantation in a murine lung model 83. This early infiltration is driven by damage-associated molecular patterns (DAMPs) released from dying cells in ischemic tissues, which trigger sterile inflammation. Inflammatory cell death, such as necrosis, necroptosis, pyroptosis, and ferroptosis, activates innate immunity and promotes the production of cytokines and chemokines that enhance neutrophil recruitment. Among these key mediators, Toll-like receptor 4 has been identified as a critical sensor of DAMPs and plays a central role in neutrophil trafficking into multiple transplanted organs, including the lung, liver, heart, and kidney 84, 85.

Surgical intervention, the most common clinical operation, provokes a robust sterile wound characterized with an increase in the level of DAMPs, which drives rapid neutrophil infiltration to the wound site within hours post-incision 86, 87. In the early stages of surgical trauma, a variety of damaged cells release DAMPs, such as ATP, formylated peptides, heat shock proteins, and pro-inflammatory cytokines, including IL-1β, TNF-α, and others 88. These cytokines interact with chemokine receptors on the surface of neutrophils to promote their migration towards the surgical site.

In ischemic stroke, neuronal necrosis leads to the release of DAMPs such as HMGB1 and ATP, which activate neurons, glial and parenchymal cells, microglia, and astrocytes to secrete IL-1β, TNF-α, inducible nitric oxide synthase, and neutrophil-attracting chemokines. These mediators promote endothelial activation and upregulation of adhesion molecules, facilitating the recruitment of neutrophils and other peripheral leukocytes from the bloodstream into the brain. Chemokine gradients within the ischemic penumbra guide neutrophils toward the infarcted region, where they contribute to both debris clearance and secondary tissue injury 80, 89-91.

### 3.3 Targeting sterile chronic inflammation

Chronic inflammation arises when the initial damage persists or the acute response fails to terminate in the resolution phase, resulting in a low-grade, sustained inflammatory state. In chronic lesions, persistent chemokine signaling drives continuous neutrophil recruitment. Neutrophil-derived proteases, extracellular traps, and cytokines then amplify the recruitment and activation of monocytes, macrophages, and lymphocytes, perpetuating the inflammatory cycle. Neutrophil accumulation has been observed in diseases such as atherosclerosis, chronic obstructive pulmonary disease (COPD), and inflammatory bowel disease 92, 93. Elucidating neutrophil migration in these contexts can provide great insights into the design of NDDSs with enhanced targeting precision.

In atherosclerosis, a prototypical chronic inflammatory disease in medium- and large-sized arteries, subendothelial lipid accumulation and immune cell infiltration lead to intimal lesions covered by smooth muscle cells and collagen. Early endothelial activation, driven by oxidative stress, proinflammatory cytokines, and oxidized lipids, upregulates adhesion molecules such as VCAM-1, ICAM-1, and E-selectin 94. Concurrently, activated endothelium releases neutrophil-attracting chemokines, including CXCL1, CXCL2, and CXCL8, which form chemotactic gradients that direct circulating neutrophils to adhere, transmigrate, and accumulate in the intima 95. Once recruited, neutrophils degranulate by releasing proteases and reactive oxygen species to degrade extracellular matrix, and their secreted mediators further amplify local inflammation by reinforcing endothelial activation and chemokine production 96. Inflammatory bowel disease (IBD) is characterized by dysregulated immune responses that drive persistent intestinal inflammation. In IBD, inflamed epithelial cells and resident immune cells produce neutrophil-attracting mediators, such as cytokines (IL-8, IL-6, IL-33), chemokines (CXCL5, CXCL7, CXCL10, CCL20), and lipid signals (leukotriene B4, hepoxilin A3), as well as matrix metalloproteinases (MMP3, MMP7) that remodel the extracellular matrix. Chemotactic gradients for these factors are established, and they engage CXCR1/2 and CCR6 on circulating neutrophils to direct their transepithelial migration into the mucosa 97. In COPD, a hallmark is neutrophilic inflammation, which is correlated with airway obstruction and small airway dysfunction. Airway epithelial cells and alveolar macrophages produce neutrophil-attracting mediators including CXCL1, CXCL5, leukotriene B4, and CXCL8 and chemotactic gradients are established within the bronchial mucosa 98. These signals engage CXCR2 on circulating neutrophils to drive their extravasation and accumulation in the airway lumen. In RA, anti-citrullinated protein antibodies bind to osteoclasts within the joint cavity to trigger the secretion of IL-8, which facilitates neutrophil recruitment. Subsequently, anti-citrullinated protein antibodies and/or the rheumatoid factor stimulate neutrophils to release NETs, amplifying local joint inflammation and promoting the accumulation of inflammatory cells in the synovium 99, 100. Effective drug delivery to inflammatory sites in the central nervous system (CNS) must surmount the challenge of crossing the blood-brain barrier (BBB), a highly selective endothelial interface that blocks the vast majority of molecules from entering neural tissues 101-104. Overcoming the barrier is particularly critical for advancing nanotherapeutic strategies for brain diseases 105-107. Neutrophils possess an intrinsic capacity to cross both the BBB and BBTB 108. In a pathological state of multiple sclerosis, experimental autoimmune encephalomyelitis, or an inflammatory TME, glial and endothelial cells secrete cytokines and chemokines (e.g., IL-8, CXCL1, and CXCL2) to activate circulating neutrophils and direct their migration toward inflamed regions 109, 110. In Alzheimer's disease models, neutrophil accumulation has been positively correlated with cognitive dysfunction. Upregulation of the ICAM-1 expression and the presence of amyloid-β (Aβ) peptides in brain lesions promote a conformational shift of LFA-1 from a closed to an open state to enhance neutrophil recruitment 108, 111-113.

### 3.4 Targeting infectious inflammation

Neutrophils, as key effector cells, initiate host defense against a range of pathogens, including bacteria and protozoa, through mechanisms such as phagocytosis, degranulation, ROS production, and NETs. These processes are tightly orchestrated to mitigate infectious threats while minimizing host tissue damage.

Neutrophil recruitment and effector function differ markedly between sterile injury inflammation and pathogen-driven inflammation. Although DAMPs are involved in both scenarios, infections uniquely introduce PAMPs, such as viral or bacterial nucleic acids, bacterial pilin, flagellin, LPS, lipoteichoic acids, or fungal carbohydrates (e.g., mannans or glucans). These PAMPs activate pattern recognition receptors on host cells, amplifying innate immune signaling and modulating neutrophil infiltration dynamics and phenotypic responses 114, 115. The recruitment process is coordinated by complex interactions among other immune cells 116. For instance, perivascular macrophages produce CXCL1 and CXCL2 to promote neutrophil extravasation at bacterial infection sites, while dendritic cells upregulate these chemokines after *Leishmania* infection 117, 118. In addition to classical antimicrobial mechanisms, NETs, web-like chromatin structures decorated with histones and granular proteins, play a pivotal role in pathogen containment. To enhance this function, Wang et al. developed engineered neutrophils preloaded with a nucleus-targeting photosensitizer characterized by high DNA affinity and potent ROS generation. Upon NETosis triggered by bacterial toxins, the nucleus-targeting photosensitizer-enriched NETs are rapidly deployed, enabling efficient bacterial entrapment and localized antimicrobial delivery 119. These findings support the therapeutic potential of harnessing engineered neutrophil responses to develop novel infection control strategies.

## 4. Strategies for Utilizing Neutrophils as Drug Carriers

**Figure 4:**
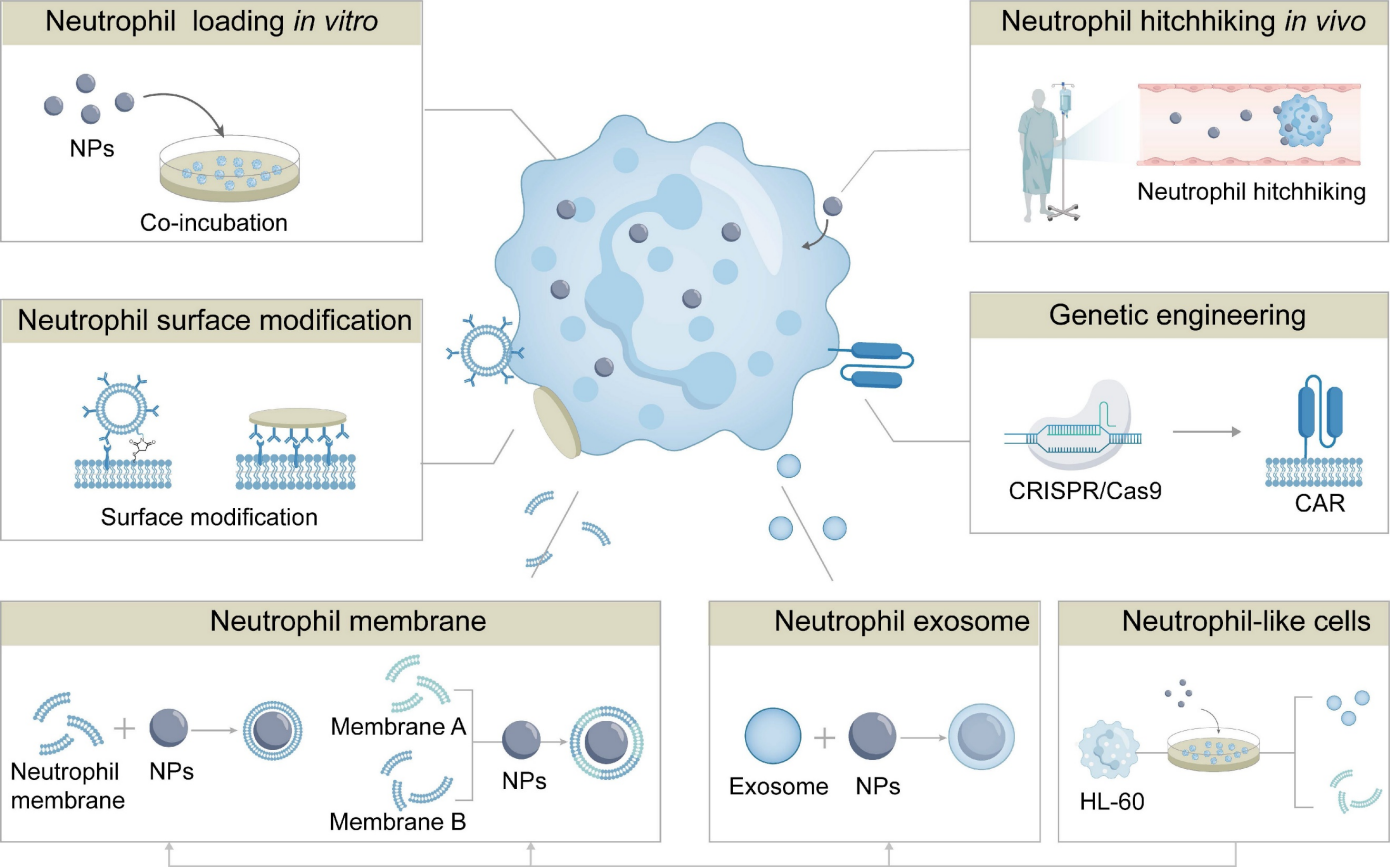
**Neutrophil-based delivery strategies.** Neutrophil-based delivery strategies include direct drug loading onto neutrophils *ex vivo*, *in vivo* hitchhiking, surface modification, and genetic engineering of neutrophils. In addition, NDDSs encompass neutrophil membrane-coated nanomedicines, hybrid membrane-coated nanomedicines, neutrophil-derived exosomes-coated nanomedicines, and neutrophil-mimicking nanoplatforms.

Neutrophil-based drug delivery strategies have been explored to enhance both the efficiency and safety of this delivery system. Depending on the approach of loading drugs into neutrophils, these strategies are categorized into *ex vivo* intracellular loading, and *in vivo* hijacking (**Figure 4**). The examples for these neutrophil-based drug delivery strategies have been listed in **Table 1**. We will elaborate the mechanisms, advantages, and limitations of these approaches for NDDSs in the following sections.

### 4.1 *Ex vivo* drug loading into neutrophils

**Figure 5:**
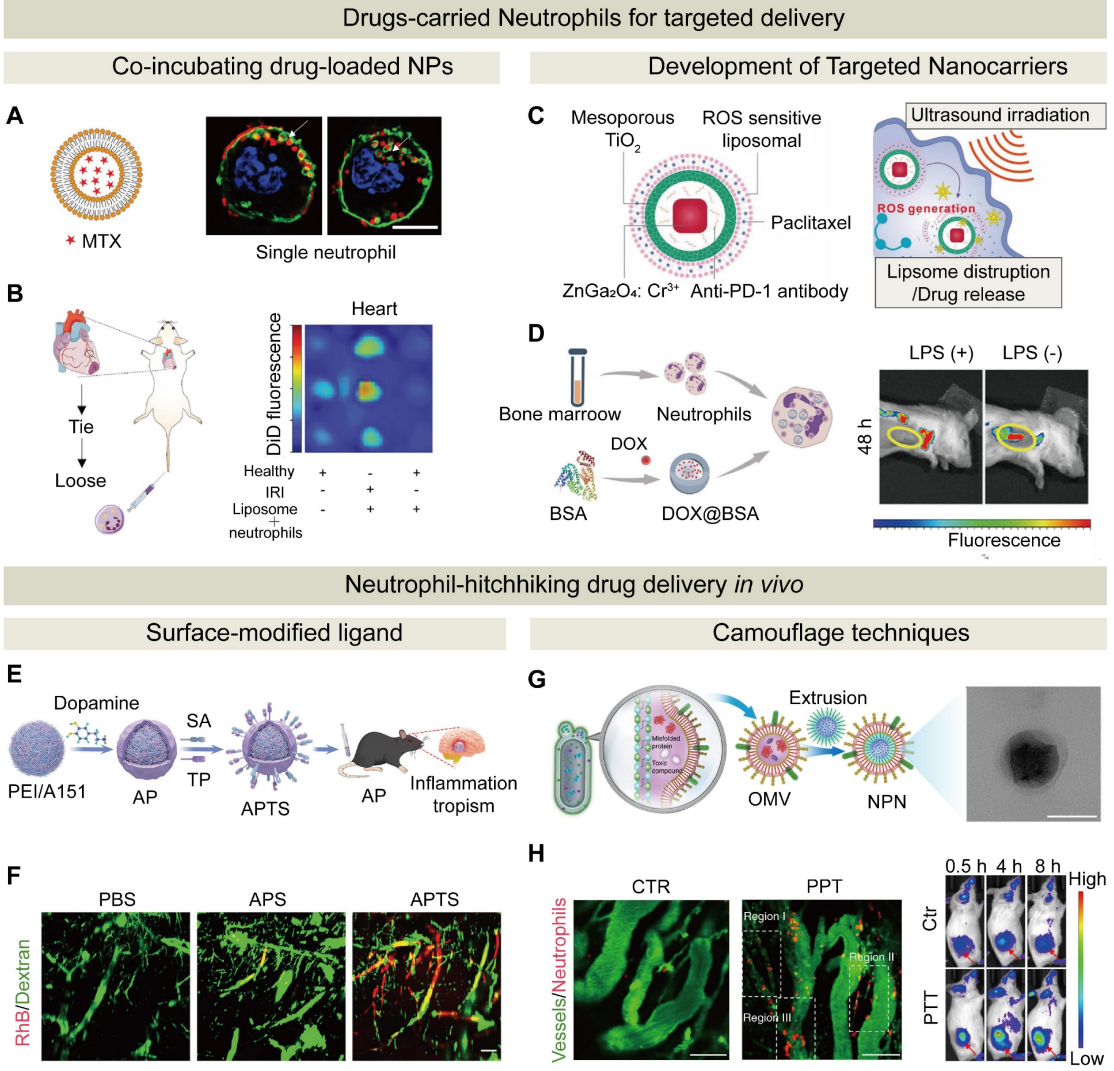
**The strategies for loading drugs into neutrophils include *in vitro* loading and *in vivo* hitchhiking.** (A) Characterization and super-resolution imaging (SIM) of MTX-liposome-loaded neutrophils, confirming liposome localization on/in neutrophils. Scale bar: 5 µm. (B) Neutrophil-mediated delivery of liposomes to the ischemic heart post-IRI. Schematic of surgical procedure and injection of DiD-labeled liposome-loaded neutrophils, and *in vivo* fluorescence images of liposomes in the heart. Adapted with permission from 123, copyright 2020 Wiley-VCH GmbH. (C) Composition of ZGO@TiO₂@APL, and ultrasound-triggered drug release in GBM. Upon insolation, ROS generation facilitated the release of PTX for therapeutic action and anti-PD-1 for sustained tumor targeting. Adapted with permission from 134, copyright 2021 Wiley-VCH GmbH. (D) Schematic synthesis of NDDSs and *in vivo* fluorescence images of mice after intravenous injection of NDDSs in non-LPS-treated and LPS-treated 4T1 tumor-bearing mice. Adapted with permission from 135, copyright 2025 Wiley-VCH GmbH. (E) A neutrophil-hijacking nanoplatform for ischemic stroke therapy. (F) Light-sheet microscopy images for vascular distribution. Adapted with permission from 144, copyright 2025 Wiley-VCH GmbH. (G) Preparation of pathogen-mimicking nanoparticle via OMVs-coated NPs to retain PAMPs and TEM image of NPs@PBT (scale bar: 100 nm). (H) *In vivo* imaging of neutrophil infiltration in tumors pre- and post-PTT (left). Scale bar: 100 µm. Fluorescence-based *in vivo* imaging at different time points (right). Adapted with permission from 246, copyright 2020 Springer Nature.

Neutrophils can be loaded *ex vivo* with NPs through co-incubation, and their innate homing property allows transporting therapeutic agents in NPs by neutrophils to pathological sites and releasing them selectively. Therefore, *ex vivo* loading into neutrophils with therapeutic agents or drug-loaded NPs, followed by their injection into patients, has been extensively explored for targeted drug delivery to distant pathological sites 120. Studies have shown that the loading process has no significant impact on neutrophil viability, apoptosis, or activation 121, 122. For instance, Stevens et al. developed a NDDS by encapsulating immunosuppressive drugs within liposomes and subsequently incubating liposomes with neutrophils *ex vivo* (**Figure 5**A). The drug-loaded neutrophils were then intravenously injected into mice, leveraging their natural homing ability to deliver the therapeutic payload to inflamed sites, such as ischemia-reperfusion areas in skeletal and cardiac muscle (**Figure 5**B). This bio-inspired strategy, which employed live neutrophils as active carriers, demonstrated efficient targeted delivery without impairing neutrophil function 123.

To load nanocarriers into neutrophils *ex vivo*, neutrophils are cultured with drug-loaded nanoparticles, which are internalized through phagocytosis. Subsequently, NPs-loaded neutrophils are harvested via centrifugation. The drug loading efficiency is quantified either by measuring the residual drug in the supernatant or lysing neutrophils to assess the intracellular drug content. The efficiency of this approach is dependent on NP physicochemical properties, such as size, shape, surface roughness, positive surface potential, and concentration 124-127. It has been discovered that neutrophils rapidly internalize NPs, and the internalization process becomes stabilized within 15 min. They display a preference for large nanoscale particles (up to 200 nm) and rod-shaped NPs. Rod-shaped NPs are internalized at a rate 3.5 times faster than sphere-shaped NPs, which is due to enhanced phagocytosis of rough or elongated structures 121, 128. Additionally, an increase in the NP concentration (e.g., from 0.1 to 3 mg/mL) could boost the proportion of drug-loaded neutrophils from 35% to 99%, which was demonstrated with improved delivery of Poly(lactic-co-glycolic acid) (PLGA) NPs and their sustained release 24. In another study, by utilizing the migration characteristics of neutrophils to postoperative inflammatory brain sites, a neutrophil platform was constructed to carry PTX liposomes. Precise targeting of residual glioma cells after surgery was achieved, resulting in effective delay in tumor recurrence and pronounced improvement in the survival rate of the animals 68. Since dysfunction of sarcoplasmic reticulum Ca²⁺-ATPase is a key driver of calcium overload that leads to oxidative stress and cardiomyocyte injury in myocardial ischemia-reperfusion injury, Jiang et al. developed PLGA-based nanoparticles to encapsulate luteolin, an activator for the sarcoplasmic reticulum Ca²⁺-ATPase, and functionalized the nanoparticles with p-toluenesulfonamide for precise sarcoplasmic reticulum targeting. By harnessing neutrophil chemotaxis and NET-mediated release, these nanoparticles were delivered to inflamed myocardial tissues via a neutrophil-hitchhiking strategy. This approach effectively restored sarcoplasmic reticulum calcium homeostasis, attenuated oxidative damage, and improved cardiac function in a myocardial ischemia-reperfusion injury model 129.

Neutrophils are short-lived immune cells, with a lifespan of approximately 19 hours. As they become aged, they undergo phenotypic changes characterized by upregulation of CXCR4 and downregulation of CXCR2. This shift facilitates their homing back to the bone marrow, where they undergo apoptosis, a process regulated by the CXCR4/CXCL12 signaling axis 130-132. Building upon this natural homing mechanism, Luo et al. developed an innovative drug delivery strategy to leverage senescent neutrophils to transport therapeutics directly to the bone marrow. In this approach, PLGA nanoparticles with encapsulated cabazitaxel or teriparatide were incubated *ex vivo* with neutrophils. These drug-loaded neutrophils were then reintroduced into the systemic circulation. Due to the innate property of aged neutrophils to return to the bone marrow, this method achieved targeted delivery of therapeutics, enhanced the local drug concentration and improved therapeutic efficacy in the models of bone metastasis and osteoporosis 24.

Despite a robust drug-loading capacity of neutrophils, predominant reliance on passive phagocytosis for drug loading into neutrophils* in vitro* may result in a low targeting efficiency, prompting the development of carriers with enhanced active targeting capabilities. Notably, in one study neutrophils were utilized to deliver CD11b-conjugated microbubbles, and this “new-balloon” system successfully released miR-126a-5p into atherosclerotic plaques. Conventional microbubbles mimic red blood cell rheology to concentrate centrally during blood flow to reduce adhesion, while these neutrophil-loaded microbubbles harnessed the unique characteristics of neutrophils to enhance targeting of microbubbles to lesion sites and strong anti-inflammatory effects were achieved through this “new-balloon” system 133. In another study, a NDDS was developed for glioblastoma (GBM) using a nanoplatform with a ZnGa₂O₄/Cr³⁺ core and a TiO₂ shell. The PTX and anti-PD-1 antibody-encapsulated nanoplatform was loaded into liposomes and engulfed by neutrophils. After injection of liposome-loaded neutrophils, neutrophils delivered the liposomes to the GBM site. Upon reaching the site, ultrasound activated the TiO₂ shell of the nanoplatform to release PTX and anti-PD-1, killing tumor cells and boosting immune responses (**Figure 5**C) 134. In addition to serving as carriers for therapeutic agents, neutrophils can be used to deliver imaging agents. For example, inflammation-responsive neutrophils were employed to internalize DOX-loaded magnetic mesoporous silica NPs for targeted glioma therapy. These NPs-loaded neutrophils actively homed to the inflammatory post-surgical tumor sites, and simultaneous drug delivery and magnetic resonance imaging were achieved. This strategy significantly enhanced intratumoral drug accumulation and effectively delayed glioma recurrence, and it could be a promising approach for integrated cancer theranostic 25.

To enhance infiltration of NDDSs, an inflammatory environment could be created at the target site. For example, DOX-encapsulated BSA NPs were loaded into neutrophils to target tumor inflammation. Before administration of this NDDS, LPS was locally injected to stimulate tumor inflammation. Compared with the mice that did not receive LPS injection, the fluorescence intensity of the drug in the mice that received LPS injection was enhanced significantly at 4 and 8 h post injection (**Figure 5**D). Therefore, the induced inflammation enhanced recruitment of drug-loaded neutrophils to the tumor site, thus improving the therapeutic efficacy 135. In addition, certain microenvironments after therapeutic treatment could promote neutrophil recruitment. For instance, Zhang et al. demonstrated that modest radiotherapy induced the release of inflammatory factors to trigger the formation of NETs. Localized and potent anti-tumor effects were realized through precise delivery of neutrophils via their homing ability and the NET formation under inflammatory conditions 136. Moreover, a PDT-induced inflammatory environment was observed to promote neutrophil recruitment 137.

The advantage of *ex vivo* loading of drugs into neutrophils lies in precise control of drug loading into neutrophils before injection and release from neutrophils upon reaching the target site. However, the *ex vivo* loading approach has encountered challenges including a short half-life of neutrophils, off-target effects due to drug release during circulation, and risks of pulmonary complications (e.g., hypoxia, hypotension) after large-scale infusion. These challenges have hampered its broad application. To enhance the stability of NDDSs, several strategies can be considered, such as simplifying drug-loading procedures to minimize the *ex vivo* manipulation time, improving biocompatibility of engineering materials to mitigate their stress on cells, and supplementing nutrient-rich media or protective agents during transportation to preserve neutrophil viability and functionality.

### 4.2 *In vivo* neutrophil hitchhiking

Compare with extracorporeal loading, *in vivo* neutrophil hitchhiking of drug-loaded NPs helps simplifying the preparation process, reducing the cost, and minimizing systemic side effects. Neutrophil hijacking for *in vivo* drug delivery involves specific engulfment of NPs carrying therapeutic agents by circulating neutrophils in the bloodstream. After the NPs are internalized by neutrophils, neutrophils can release the drug for direct action, alternatively, they are redirected to infection or inflammation sites through their innate targeting ability, achieving targeted drug delivery at the disease site. Compared to traditional drug carriers or other cells-based drug delivery systems, NDDSs exhibit distinct kinetic characteristics, and they can effectively deliver drugs to ischemic areas even under conditions of low blood flow or vascular occlusion 138.

Generally, the feasibility of this *in vivo* neutrophil hijacking approach relies on the engulfment of NPs by circulating neutrophils. A high drug delivery efficiency could be achieved by a high drug loading capacity into a large quantity of neutrophils. Strategies have been developed including functionalizing the nanoparticle surface with neutrophil-targeting antibodies or aptamers, or coating the NPs with exogenous functional moieties to enhance the surveillance capability of neutrophils 75. Through these strategies, neutrophils could be effectively utilized as vehicles for targeted drug delivery into the inflammatory sites. Specific peptides on NPs have been explored for targeting neutrophils. The cRGD peptide that binds to integrin αvβ1 exhibits high affinity for neutrophils and monocytes. Hou et al. confirmed high binding affinity of cRGD peptide to the targeted receptor αvβ1 via surface plasmon resonance. The uptake of liposomal drugs by neutrophils was enhanced through surface modification of liposomes with the cRGD peptide. Inflammatory responses after ischemic injury induced the recruitment of white blood cells (predominantly monocytes and neutrophils) to the infarct core and penumbra, thus liposome-loaded neutrophils successfully delivered edaravone in the liposomes to the ischemic subregions in the brain, which provided a unique therapeutic intervention opportunity for the ischemic brain disease 138. Another nanosystem that consisted of a LiMn₂O₄ nanozyme core mimicking superoxide dismutase (SOD), catalase (CAT), and glutathione peroxidase (GPx) activities and a cRGD-modified liposomal shell was prepared for internalization by circulating neutrophils. After the nanosystem-loaded neutrophiles reached the inflammatory site, the nanozyme core was released to scavenge ROS, reduce oxidative stress, and inhibit apoptosis, effectively mitigating acute kidney injury -related damage 139. Tang et al. conjugated a neutrophil-selective binding peptide (CFLFLF) to a ROS-responsive polymer. A therapeutic drug GSK484 was encapsulated into the polymer to form NPs. After intravenous administration of the NPs, they encountered and entered circulating neutrophils. The neutrophils rapidly travelled to the site of brain injury and released the NPs at the injury site. Due to an elevated ROS level at the injury site, the nanoparticle shell was degraded to release GSK484. This drug, in turn, inhibited the formation of NETs, thereby reducing microglial and astrocyte-induced neuroinflammation 140. In a recent study, a nucleic acid-based nanoplatform with a tetrahedral framework was functionalized with Ac-PGP, a CXCR2-targeting peptide that enables specific recognition and uptake by circulating neutrophils. This modification allowed this system to effectively “hitchhike” on neutrophils to inflammatory sites. *In vivo*, this strategy significantly enhanced the bioavailability of baicalin and modulated immune responses by promoting macrophage polarization from a pro-inflammatory M1 phenotype to an anti-inflammatory M2 phenotype, thereby alleviating systemic inflammation in a sepsis model 141.

In addition to peptides that can target neutrophils, antibodies, polysaccharides and other ligands can bind to specific receptors on the surface of neutrophils. For instance, gold nanorods conjugated with anti-CD11b antibodies were employed as a therapeutic nanomedicine (NPs-CD11b) for cancer treatment. The combination of 660 nm light and pyrophosphate-a (Ppa) was used to induce photosensitization, which triggered inflammation and encouraged neutrophil infiltration into the tumor site. NPs-CD11b was internalized by activated neutrophils after administration. Subsequently, NPs-CD11b-carried neutrophils infiltrated into the tumor tissue, resulting in the accumulation of NPs-CD11b in inflammatory tumors 35 times higher than that of NPs-PEG without neutrophil hitchhiking. By contrast, tumor accumulation of NPs-CD11b was significantly decreased in inflammatory tumors after systemic neutrophil depletion 142. Wu et al. developed a tannic acid nanoparticle-based system that was surface-functionalized with an anti-Ly6G antibody and interferon-β. By targeting neutrophils via the Ly6G antibody, the system “hitchhiked” on neutrophils to achieve precise delivery to inflamed sites in the CNS. Upon neutrophil arrival and NET release at the inflammatory site, the nanoparticles release interferon-β to effectively modulate immune responses, reduce inflammation, and improve neurological function 143. Another neutrophil-hijacking nanoplatform was developed by modifying NPs with a targeting peptide (TP) and sialic acid (SA) on a polydopamine coating. The resulting nanoplatform achieved efficient oligonucleotide delivery and neuroinflammation modulation. The TP served as a docking point for transglutaminase, which is overexpressed on inflamed endothelial cells, facilitating neutrophil recognition and hijacking. Meanwhile, SA enhanced neutrophil uptake by binding to L-selectin on activated neutrophils (**Figure 5**E). The imaging results of light-sheet microscopy revealed that, compared with the NPs that were not modified with SA, the NPs modified with SA significantly enhanced their accumulation in tumor blood vessels (**Figure 5**F). After reaching the tumor site, this nanoplatform reprogramed neutrophil death from NETs to apoptosis through a ROS scavenging-mediated histone citrullination inhibition pathway, thereby suppressing excessive neuroinflammation by blocking NET release. This study demonstrated a promising approach for targeted drug delivery to the brain and immune microenvironment regulation to treat neuroinflammatory diseases 144.

Apart from surface-modified antibodies to enhance neutrophil targeting, another strategy for *in vivo* neutrophil hitchhiking involves the use of camouflage techniques. To mimic the process of phagocytosing bacteria by neutrophils, NPs could be disguised with bacterial moieties to facilitate their “phagocytosis” by neutrophils *in vivo*. Zhou et al. prepared a pathogen-mimicking nanoparticle for treating ischemic stroke by coating of pioglitazone with bacterial-derived outer membrane vesicles (OMVs). OMVs as a bacterial component could significantly enhance neutrophil uptake of the NP. Pathogen-mimicking nanoparticle-internalized neutrophils penetrated through the BBB and homed to the ischemic brain regions. Upon reaching the ischemic area, excessive ROS produced at the ischemic region promoted the dissociation of neutrophil chromatin, leading to the formation of NETs. Pioglitazone was released during the formation of NETs to reduce the level of IL-1β by inhibiting the aggregation of the NLRP3 inflammasome 145. Wang et al. constructed a pathogen-mimicking nanoparticle system by coating NPs with OMVs to achieve active tumor targeting by efficient neutrophil hijacking (**Figure 5**G). Through intravital microscopy and an *in vivo* imaging system, they demonstrated that photothermally triggered inflammation synergistically enhanced neutrophil infiltration into the TME, thereby improving therapeutic efficacy (**Figure 5**H). Complement proteins could also be employed for surface camouflaging of the delivery system. The complement system is the first line of defense against pathogens in the body, and it can rapidly recognize and eliminate foreign invaders. Interestingly, some pathogens exploit the complement system to facilitate “self-modulation” through the complement fragment iC3b, which enhances phagocytosis via the complement receptor 3 (CR3). By fine-tuning the chemical composition of NPs, Wang et al. prepared a protein corona that was spontaneously bound to specific blood proteins. After the complement fragment iC3b accumulated to molecularly engineered liposomes with phosphocholine lipids, neutrophil uptake of the liposomes was triggered via CR3-mediated phagocytosis. Neutrophils that carried the drug-loaded liposomes migrated across the alveolar-capillary barrier to the inflammatory tissue. They either released the liposomes through the formation of NETs or acted as micro-containers to sequester and kill bacteria, thereby controlling infection 146. Platelet membrane proteins (e.g., CD47, CD62P) can also be utilized for coating NPs since they can be recognized by activated neutrophils, thus these modified NPs can be delivered to a targeted inflammation site via a “hitchhike” mechanism. For example, a platelet membrane-mimicking nanoparticle was prepared. The nanoparticle bound to activated neutrophils during circulation, and it was delivered to the targeted site with the help of neutrophils. The embedded nanozyme (PtCD) scavenged neutrophil-derived ROS to reduce oxidative damage, while the inhibitor piceatannol blocked neutrophil adhesion and aggregation to promote their clearance and significantly block their infiltration (from 60.8% to 8.21%). This dual-action strategy could be promising for ulcerative colitis treatment 147. Wang et al. developed nanoparticles with a PLGA core cloaked with hybrid membranes from platelet-derived extracellular vesicles with expressed P-selectin and membrane from doxorubicin-pretreated L929 cells (a mouse fibroblast-like cell line) with calreticulin. These nanoparticles leveraged P-selectin to target activated neutrophils, and they homed to inflamed tissues via neutrophil migration. Calreticulin expressed on the L929 cell surface induced by doxorubicin acted as an exogenous “aged” signal to trigger macrophage-mediated premature programmed cell removal. This approach effectively relieved inflammation and prevented tissue damage in acute lung injury and severe acute pancreatitis models by clearing activated neutrophils 148.

In clinical application, *ex vivo* drug-loading allows precise control over dosage and ensures uniform drug loading into each neutrophil, thus this approach is applicable for therapies with high doses or defined release kinetics. However, isolation of neutrophils from patients may temporarily deplete endogenous neutrophils, and reinfusion of these cells back to patients can induce cytokine storms or pulmonary complications. In contrast, *in vivo* hijacking is less invasive and it harnesses the natural ability of neutrophils to home to inflamed or infected sites for targeted delivery of drugs to these sites. This method is particularly applicable for acute inflammation or infection where neutrophils rapidly mobilize to disease sites. However, this strategy requires great efforts into nanoparticle design, particularly for selectively targeting activated neutrophils during circulation while minimizing off-target uptake by other cells, which are essential for safety and therapeutic efficacy.

## 5. Engineering Neutrophils for Enhanced Therapeutic Function

Intracellular uptake of NPs by neutrophils inevitably impairs their activity and induces a shift in their phenotypes. To address this, two safe and non-invasive engineering strategies have been developed: (1) surface functionalization of neutrophils enables the attachment of targeting ligands, antibodies, or biomimetic coatings onto the cell surface, thereby promoting efficient adhesion and directed migration to specific lesions; (2) non-integrating genetic engineering through mRNA transfection, a transposon system, or a CRISPR-Cas9 ribonucleoprotein delivery system allows transient expression of therapeutic proteins or receptor molecules or their precursors on or in neutrophils. The examples for these engineering neutrophils strategies have been listed in **Table 1**. These molecules endow engineered neutrophils with programmable special functions, such as controlling the N1 phenotype of neutrophils, or secreting specific cytokines to improve the treatment effect (**Figure 4**).

### 5.1 Surface engineering for improved targetability and functionality

**Figure 6:**
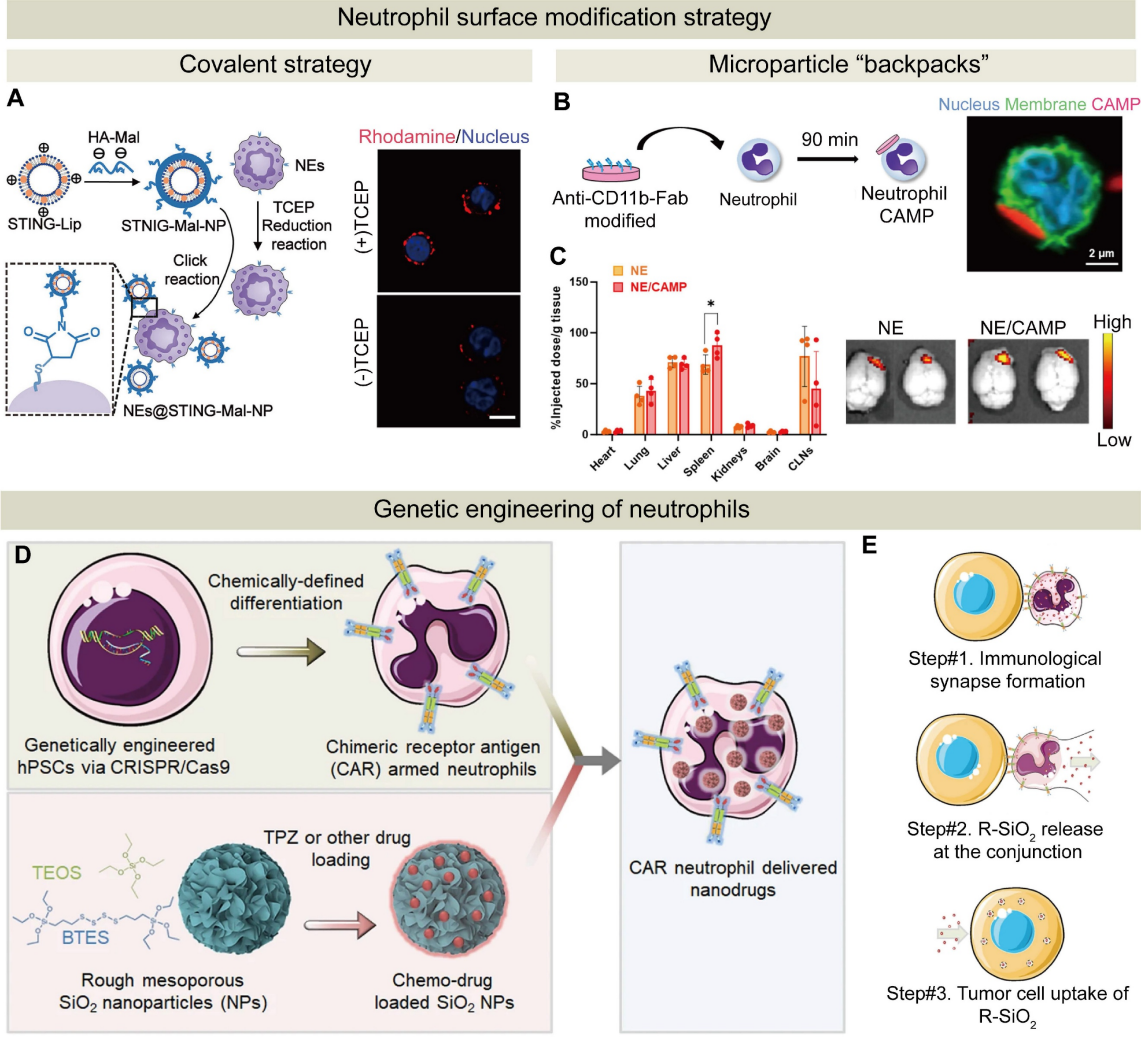
**The strategies of surface modification and genetic engineering of neutrophils.** (A) Schematic of NEs@STING-Mal-NP preparation via reduction-click chemistry. Confocal images confirmed that STING-Mal-NP-RhoB (red) bound to neutrophils with or without tris (2-carboxyethyl) phosphine treatment. Nuclei stained with Hoechst (blue). Scale bar: 5 μm. Adapted with permission from 69, copyright 2023 American Chemical Society. (B) Preparation of NE/CAMPs by incubating anti-CD11b-Fab-conjugated CAMPs with primary NEs. Confocal images supported NEs with surface-bound CAMPs. (C) Biodistribution of NE/CAMPs in orthotopic GL261 glioma mice at 24 h post-injection. Images from an* in vivo* imaging system confirmed increased brain accumulation of NE/CAMPs versus the control NEs. Adapted with permission from 26, copyright 2024 John Wiley and Sons. (D) CAR-engineered hPSC-derived neutrophils loaded with a hypoxia-targeting drug (tirapazamine) via rough SiO₂ (R-SiO₂) NPs for dual immunochemotherapy. (E) Polarized F-actin accumulates at the interface between CAR-neutrophils and tumor cells, followed by the release of R-SiO₂-tirapazamine nanoparticles upon phagocytosis, which are subsequently internalized by the tumor cells. Adapted with permission from 28, copyright 2023 Springer Nature.

Neutrophil surface modifications can be achieved through the formation of stable chemical bonds, such as amide bonds or maleimide-thiol addition 149. For example, a neutrophil-based delivery system was designed by conjugating STING agonist-loaded liposomes onto neutrophils via a thiol-maleimide click reaction. In this approach, the liposomes were functionalized with maleimide groups by coating them with hyaluronic acid-maleimide (HA-Mal), while neutrophils were treated with tris (2-carboxyethyl) phosphine to expose thiol groups on the surface. The resulting covalent linkage enabled stable attachment of the NPs to the neutrophil membrane, facilitating targeted delivery of a STING agonist to tumors (**Figure 6**A). Neutrophil extravasation and infiltration into tumors led to a significantly improved level of tumor penetration and accumulation of the STING agonist in triple-negative breast cancer (TNBC) 69.

Covalent modifications of neutrophils may compromise the drug activity, impair functions of neutrophils including migration and phagocytosis, and introduce potential toxicity. Non-covalent modifications of neutrophils with short lifespans become popular due to their mild and reversible nature 149. A novel technique for non-invasive modification of neutrophil cell surfaces is developed using microparticle “backpacks” that adhere to the surface of neutrophils, modulating their function without internalization by neutrophils. These structures are designed to modulate the cellular behavior, specifically regulating the neutrophil state. Neutrophils can display multiple physiological states. For instance, neutrophils, similar to macrophages, can be in a pro-inflammatory or anti-inflammatory state. In the TME, they are often regulated by secreted factors from tumors to switch from a tumor-killing state to a tumor-promoting one. Kumbhojkar et al. developed a novel backpack, which was termed as Cyto-Adhesive Micro-Patches (CAMPs). These disc-shaped microparticles were composed of a mixture of two polymers and they successfully attached to the surfaces of neutrophils using an antibody fragment on two polymers to target a specific protein on the cell surface. Neutrophils were polarized into an anti-tumor phenotype after adherence to disc-shaped polymeric micro-patches on their surface without internalization of them. *In vivo* imaging confirmed that intravenously injected neutrophils with micro-patches primarily accumulated in the spleen and tumor-draining lymph nodes, where they activated splenic NK cells and T cells and enhanced the accumulation of dendritic cells and NK cells. In a tumor mouse model, micro-patch-activated neutrophils, in combination with a checkpoint inhibitor, anti-cytotoxic T-lymphocyte-associated protein 4 (anti-CTLA-4), effectively activated the immune checkpoint to suppress tumor immune evasion, resulting in complete tumor regression in one third of the treated mice 150. In another similar study, CAMPs were designed to allow specific binding of anti-CD11b Fab fragments on CAMPs to CD11b on the neutrophil surface (**Figure 6**B). By attaching these CAMPs to neutrophils, they mechanistically induced the switch of neutrophils to an anti-tumor phenotype, thereby enhancing their capacity to cross the BBB and promoting the aggregation of drugs in the brain (**Figure 6**C) 26.

In addition to modification of neutrophils with large-sized backpacks, regulating cell surface glycoRNAs could be another approach to modulating the neutrophil function. Extracellular RNA species have been identified on the neutrophil surface, including a subset modified by glycosylation which is referred to glycoRNAs 151. These glycoRNAs are primarily localized on the cell membrane and they play a crucial role in mediating neutrophil adhesion and transmigration through interaction with endothelial cells. Their depletion significantly diminishes the number of neutrophils recruited to inflammatory tissues. Notably, glycoRNAs are recognized by P-selectin 152. Therefore, leveraging the natural role of glycoRNAs to strengthen neutrophil-endothelial interaction could provide a novel and physiologically relevant strategy for neutrophil surface engineering and functional modulation.

### 5.2 Genetic engineering to modulate neutrophil behavior

Neutrophils can be modified to deliver specific therapeutic agents to targeted sites via genetic engineering. Gene editing allows for precise programming of neutrophils to synthesize and deliver therapeutic agents to inflammatory tissues. Genetic engineering of neutrophils allows therapeutic proteins to be intracellularly synthesized after the genetic information is delivered into neutrophils via electroporation or gene delivery vectors (e.g., viral vectors, lipids, or polymers). The therapeutic protein is subsequently released at inflammatory sites to achieve its function. However, gene editing of neutrophils faces many challenges: (1) Neutrophils, highly specialized immune cells, exhibit an inherently low efficiency in uptake and transfection of exogenous gene. Conventional methods like electroporation often compromise the cell viability, further diminishing the genetic engineering efficacy; (2) Their short lifespan creates critical temporal constraints for sustained gene editing; and (3) Immune recognition of viral vectors or exogenous genetic materials can trigger adverse reactions.

To overcome these challenges, Chang et al. successfully engineered human pluripotent stem cells with synthetic CARs. They differentiated into highly efficient neutrophils using a chemically unique platform. The resulting chlorotoxin-targeting CAR neutrophils exhibited a typical neutrophil phenotype but selectively targeted GBM tumor cells via membrane-associated MMP2 binding. Compared to wild-type neutrophils, chlorotoxin-targeting CAR neutrophils significantly inhibited tumor growth and prolonged survival in an orthotopic GBM xenograft model 153. Building on this study, they subsequently optimized the neutrophil functionality. Human pluripotent stem cells (hPSCs) were engineered using CRISPR-Cas9-mediated gene knock-in to express various anti-glioblastoma CAR constructs with either a T-cell-specific CD3ζ or a neutrophil-specific γ signaling domain 28. Importantly, the use of hPSCs facilitated easy genomic editing, and these hPSCs were expanded and differentiated into a substantial number of neutrophils. CAR-neutrophils differentiated from the CAR-expressing hPSCs maintained an N1 anti-tumor phenotype and exhibited enhanced anti-glioblastoma activity under a hypoxic TME condition. Moreover, a biodegradable mesoporous organosilica nanoparticle with a rough surface was utilized to load a chemotherapeutic drug tirapazamine or temozolomide and the JNJ-64619187 compound. The NPs were internalized into CAR-neutrophils differentiated from hPSCs, and the resulting drug delivery system displayed superior anti-glioblastoma activity (**Figure 6**D). CAR-neutrophils released R-SiO_2_-tirapazamine NPs during tumor cell phagocytosis, and these NPs were subsequently uptaken by tumor cells (**Figure 6**E). Meanwhile, analysis of the silicon content indicated that 20% of the nanomedicine was delivered to the brain tumor through CAR-neutrophils, which significantly exceeded the delivery efficiency of less than 1% typically achieved by conventional nanomedicines via the circulatory system 28.

## 6. Biomimetic Delivery Systems Inspired by Neutrophils

Although encouraging results of neutrophils-based drug delivery systems have been demonstrated in a variety of animal models, critical issues such as yield, safety, and quality control must be addressed before clinical translation. Neutrophils-mimicking drug delivery strategies have been developed by utilizing neutrophil membranes, neutrophils-derived exosomes, and neutrophil-like cells. These strategies exhibit several advantages (**Figure 4**): (1) both neutrophil membranes and exosomes preserve the inherent chemotactic properties of neutrophils while avoiding the complexities associated with live cell usage; (2) neutrophil-like cells possess greater operational flexibility for functional modifications to optimize targeting specificity and enhance therapeutic efficacy compared to natural neutrophils. Therefore, these neutrophils mimicking strategies could be translated into clinical application, and the examples for these strategies are summarized in **Table 2**.

### 6.1 Neutrophil membrane-coated systems

**Figure 7:**
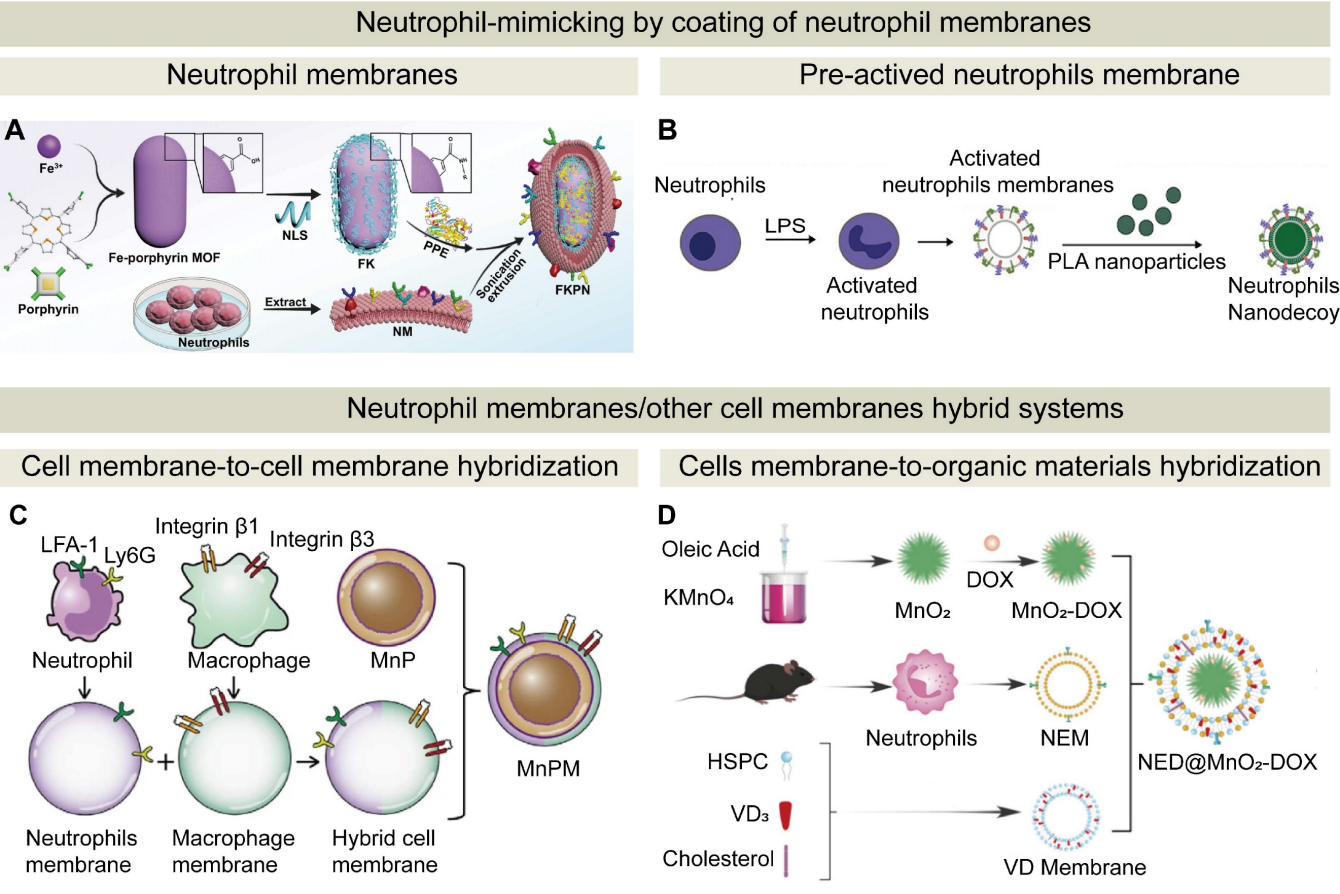
** Biomimicking drug delivery systems prepared by coating NPs with neutrophil membranes, hybrid membranes from neutrophils and other cell types.** (A) Schematic illustration of FKPN nanodevice preparation. Adapted with permission from 168, copyright 2023 Springer Nature. (B) Schematic of activated neutrophil membrane-coated NPs and their assembly. Adapted with permission from 165, copyright 2024 American Chemical Society. (C) Schematic diagram for the preparation of MnPM via fusion of neutrophil and macrophage cell membranes. Adapted with permission from 177, copyright 2024 Springer Nature. (D) The preparation process of NED@MnO₂-DOX NPs with a schematic structure and their TEM image. Adapted with permission from 179, copyright 2024 American Chemical Society.

Coating of NPs with neutrophil cell membranes has emerged as a promising surface modification strategy (**Figure 7**). This coating approach endows the NPs with enhanced bio-interfacial properties, including homogenous targeting, efficient drug delivery, immune evasion, and prolonged circulation 154-157. The neutrophil membrane retains key characteristics of surface protein markers on native neutrophils. When the membrane is coated onto nanoparticles, these membrane-derived markers can mediate specific interactions with target cells or tissues, thereby enhancing targeted delivery of therapeutics. Two typical methods for neutrophil membrane coating are extrusion and sonication. During the extrusion process, neutrophil membranes mixed with NPs pass through polycarbonate membranes with varying pore sizes to facilitate membrane fusion with NPs, while during the sonication process, neutrophil membranes are incubated with nanoparticle cores, and ultrasonic disruption promotes membrane adhesion to the NP surface. Emerging techniques such as microfluidic sonication/electroporation 158, 159 and flash nanocomplexation 160 can optimize the coating process. Neutrophil membranes as a coating layer on NPs provide a barrier against immune recognition and clearance and inherit protein receptors and adhesion factors of neutrophils. These characteristics enable specific interaction between the neutrophil membrane-coated NPs and targeted inflammatory tissues, and the neutrophil membrane-coated NPs retain the ability of crossing biological barriers including the BBB. Compared with NDDSs at a size of tens of microns, the neutrophil membrane-coated NPs are at a nanoscale, which improves their *in vivo* pharmacokinetic characteristics.

By coating a polymer core by neutrophil membranes, Zhang et al. developed neutrophil membrane-coated NPs. These NPs inherited the antigenic features and retained the membrane functions of the source cells (neutrophils), and they served as decoys for neutrophil-targeting biomolecules. These NPs were found to neutralize pro-inflammatory cytokines, inhibit synovial inflammation, penetrate deep into the cartilage matrix, and provide robust cartilage protection against joint damage 161. Kang et al. developed a nanoscale neutrophil-mimicking nanomedicine, in which the PLGA NPs core was coated with neutrophil membranes. Compared to the uncoated NPs, neutrophil membrane-coated NPs more efficiently captured circulating tumor cells (CTCs)* in vivo* and exhibited better homing to metastatic niches 27. Yang et al. investigated the intrinsic bioactivity of neutrophil membrane-coated NPs without drug loading. They demonstrated that neutrophil membrane-coated NPs could constitute a promising inflammation-regulating nanoplatform. By harnessing the targeting capabilities of activated neutrophil membranes and the anti-inflammatory signals from apoptotic membranes, neutrophil membrane-coated NPs effectively neutralized inflammatory cytokines and modulated immune cell recruitment, thereby bringing distinct therapeutic benefits in an LPS-induced liver inflammation model 162. In another study, a biohybrid microrobot was developed by conjugating *Chlamydomonas reinhardtii* microalgae onto PLGA NPs coated with neutrophil membranes via click chemistry, and the microrobot achieved targeted delivery of antibiotics for lung infection. The neutrophil membrane conferred immune evasion, prolonged circulation, and pathogen-specific adhesion by mimicking natural cell surfaces 163.

Furthermore, leveraging the targeting capability of neutrophils, neutrophil membranes have been employed to coat a variety of NPs, such as ZIF-8 cores 164, poly(lactic acid) (PLA) 165, and polyacrylamide (PAM) 166. For example, neutrophil membranes isolated from neutrophils in the mouse peripheral blood were used to coat PLGA NPs loaded with levofloxacin. The neutrophil membrane cloak endowed the NPs with lesion-targeting and mucus-penetrating capabilities, enabling efficient levofloxaci delivery to inflamed sites, and the antibiotic successfully suppressed bacterial infection 167. A biomimetic nanodevice was engineered to selectively target and eliminate cancer cells by mimicking the neutrophil functions. This device comprised a neutrophil membrane-coated Fe-porphyrin metal-organic framework (MOF) loaded with porcine pancreatic elastase (PPE) and nuclear localization signal-tagged porphyrin (porphyrin-NLS) (**Figure 7**A). Upon entering the TME, an elevated intracellular glutathione level triggered the release of PPE and porphyrin-NLS from the MOF. PPE facilitated proteolytic liberation of the CD95 death domain, and porphyrin-NLS, under laser irradiation, produced singlet oxygen to induce DNA double-strand breaks. This cascade induced by PPE and porphyrin-NLS promoted translocation of the histone H1 isoform, leading to selective apoptosis in cancer cells and stimulating adaptive immune responses for both primary and distant tumors 168. In another study, activated neutrophil membrane-coated NPs were prepared as nanodecoys to inhibit neutrophil-driven cancer metastasis (**Figure 7**B). Upon activation, neutrophils underwent morphological changes. The membranes from activated neutrophils were coated onto PLA NPs to form a core-shell structure. These activated neutrophil membrane-coated NPs effectively interfered with neutrophil adhesion to tumor endothelium and CTCs, thereby disrupting CTC-neutrophil cluster formation both *in vitro* and *in vivo* 165. Collectively, these studies have demonstrated the tremendous potential of using neutrophil membranes in biomimicking drug delivery systems.

To achieve multi-functionalities of drug delivery systems, various cell membrane fusion techniques to form hybrid membranes have been developed. Hybrid membranes for drug delivery systems integrate membranes from different cell types to optimize targeting, cellular uptake, and therapeutic efficacy 169, 170. By combining distinct membrane properties, such as immune evasion, specific ligand targeting, and enhanced stability, these drug delivery systems could improve drug bioavailability and enhance precise targeting of disease sites, particularly for cancer, inflammation, and tissue-specific diseases.

It has been shown that hybrid membranes derived from neutrophils and platelets can effectively target primary tumor cells and CTCs. Specifically, P-selectin on the platelet membrane binds to PSGL-1 or CD44 on primary tumor cells, while β-integrins on the neutrophil membrane interact with adhesion molecules such as VCAM-1 on CTCs. This interaction significantly inhibits the metastasis of breast cancer 171. Additionally, platelet-neutrophil hybrid membrane vesicles facilitated recognition and neutralization of pro-inflammatory cytokines. Upon modification with DNase I, these vesicles effectively degraded NETs, protected the blood-spinal cord barrier, and reprogramed neuroinflammatory processes, leading to promising outcomes for treating spinal cord injury in mouse models 172. A hybrid membrane derived from neutrophils and platelets inherited neutrophil-like chemotactic properties and platelet-like bacterial adhesion properties, thus the hybrid membrane-incorporated nanomedicine achieved targeted delivery to the site of inflammation induced by bacterial infection 173. In another study, a biomimetic platelet-neutrophil hybrid membrane was coated onto NPs containing R848 and CD47 to achieve potent immunomodulatory effects. The biomimetic hybrid membrane targeted inflammatory wound sites. With the assistance of photothermal stimulation, R848 was released to reprogram macrophages and enhance T-cell immunity, and the CD47 blockade amplified macrophage-mediated tumor cell clearance 174.

In one study on glioma, a novel hybrid cell membrane-coated nano-platform derived from macrophages and neutrophils was reported 175. Within a biofilm microenvironment, MnO₂ catalyzed H₂O₂ to generate O₂, boosting ultrasound-activated sonodynamic therapy (**Figure 7**C). Concurrently, Mn²⁺ release activated the cGAS-STING pathway, promoting innate and adaptive immune responses. In another study, a neutrophil-macrophage hybrid membrane was utilized to achieve precise targeting of inflammatory myocardial tissues during the early stage of acute myocardial infarction. This coordinated strategy achieved effective biofilm eradication and supported tissue regeneration 176, 177.

In addition to cell membrane-to-cell membrane hybridization, neutrophil membranes can be fused with organic materials. For instance, a high drug-loaded MSN core was coated with a neutrophil-DOTAP hybrid membrane to overcome post-ischemic cardiac inflammation and promote myocardial regeneration. By integrating precise immune modulation with targeted gene delivery, this platform effectively reprogramed the local microenvironment and stimulated cardiomyocyte proliferation 178. A neutrophil membrane and a vitamin D₃-inserted lipid hybrid shell were fused to coat MnO₂-DOX NPs, and the resulting nanoplatform, NED@MnO₂-DOX, was equipped with both immune camouflage and glioma-targeting capabilities (**Figure 7**D). This biomimetic hybrid membrane exhibited an enhancement in endocytosis of the nanoplatform, facilitated the penetration of the BBB, and achieved homing to the tumor site through interaction between inflammatory chemokines and neutrophil membranes 179. By leveraging the targeted delivery capacity, the platform initiated cGAS-STING-mediated immune activation and realized effective drug release within the glioma microenvironment.

### 6.2 Neutrophil exosome-derived systems

In addition to neutrophil cell membranes, exosomes from neutrophils have been explored for preparing biomimetic drug delivery systems. Exosomes are nanoscale extracellular vesicles that are naturally secreted by cells. They play a crucial role in intercellular communication, particularly in immune cell signaling. They contain specific proteins and nucleic acids, primarily small RNAs, such as ribosomal RNA, transfer RNA, microRNA, and mRNA molecules 180-182. The content of exosomes from neutrophils is dependent on their differentiation and polarization. Exosomes from N1 neutrophils are enriched with pro-inflammatory molecules, including miR-223 that plays a key role in myeloid cell development, defensin 1 to recruit lymphocytes, and factors like RAP1A that inhibits metastasis, along with pro-inflammatory cytokines, such as IL-1β, IL-2, and IL-4. By contrast, exosomes from N2 neutrophils contain regulatory biomolecules, including IL-6 that promotes neutrophil polarization to an N2 phenotype via the IL-6-STAT3-ERK1/2 pathway 183, 184. Therefore, strict control of neutrophil polarization during *in vitro* culture of neutrophils is essential to obtain homogeneous exosome products.

Exosomes derived from various cell types, including tumor cells, macrophages, T cells, and NK cells, have been widely explored as drug delivery carriers 185, 186. Neutrophils possess unique targetability towards inflammatory tissues, and exosomes produced from neutrophils can retain the targetability, thus they are also employed to prepare nano-delivery systems. Wang et al. reported that neutrophil-derived exosomes loaded with an agent rapidly crossed the BBB and migrated to the brain. The BBB penetration capacity and tumor targetability of neutrophil-derived exosomes were confirmed in both zebrafish and mouse brain models. In this context, neutrophil-derived exosomes hold great potential for targeted drug delivery across the BBB to the brain to treat neurological disorders 187. In addition to their targeting capability, neutrophil exosomes exhibit other physiological activities. Jiang et al. demonstrated that neutrophil-derived exosomes effectively targeted synovitis, selectively neutralized pro-inflammatory factors, and alleviated oxidative stress. Therefore, the exosomes from neutrophils exhibit targeting specificity through surface proteins and their incorporated neutrophil-derived cargos retain functional characteristics inherent from neutrophils, thereby enhancing their biological activity 188.

**Figure 8:**
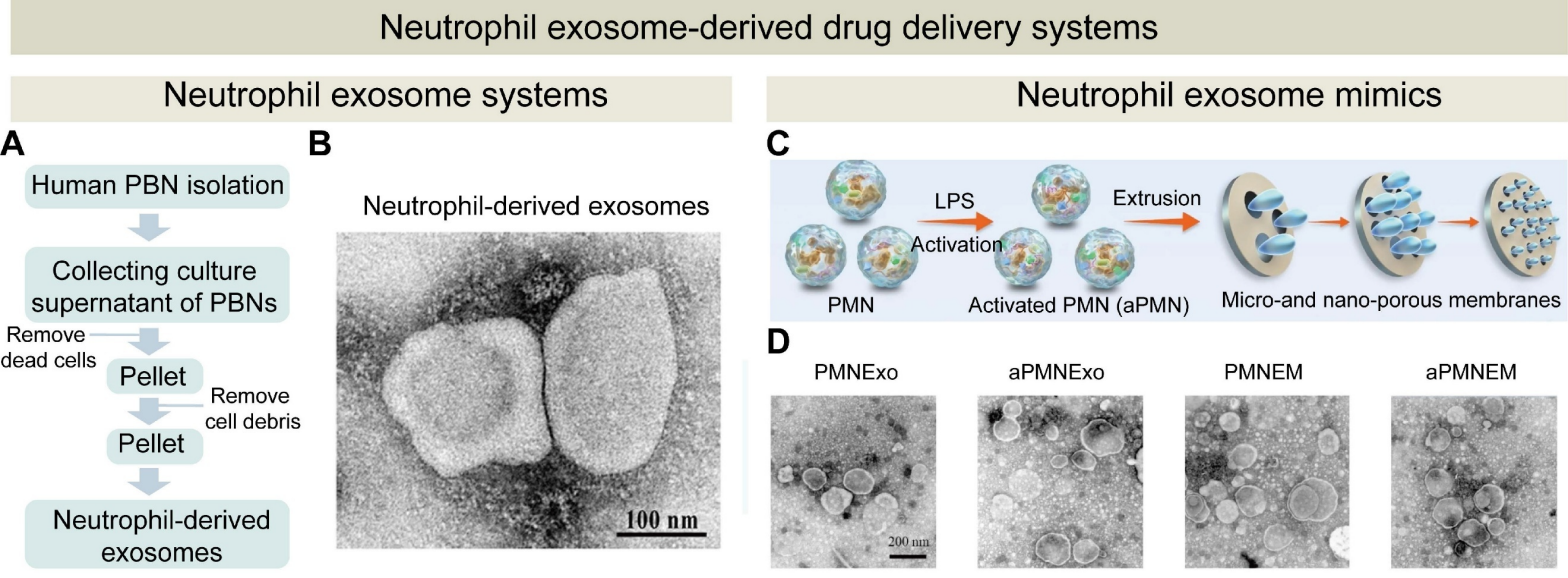
**Representative strategies for transporting drugs by neutrophil-derived exosomes.** (A) Steps for the isolation of exosomes from peripheral blood neutrophils (PBNs). (B) TEM images of neutrophil-derived exosomes. Scale bar: 100 nm. Adapted with permission from 189, copyright 2022 The American Association for the Advancement of Science. (C) The preparation of activated neutrophil exosome mimetics (aPMNEMs) via multilayer filtration of LPS-stimulated neutrophils. (D) TEM characterization of native and activated neutrophil-derived exosomes. PMNExo: Polymorphonuclear neutrophil-derived exosomes; aPMNExo: activated neutrophil-derived exosomes; PMNEM: neutrophil exosome mimetics; and aPMNEM: activated neutrophil exosome mimetics. Adapted with permission from 190, copyright 2023 Springer Nature.

The primary challenge of utilizing neutrophil-derived exosomes as drug delivery vehicles is their low yield, which can be attributed to a short lifespan of neutrophils. A low production yield of neutrophil-derived exosomes has hampered their application in cancer therapy. To address this issue, Zhang, et al developed a method to isolate neutrophil exosomes with the aid of superparamagnetic iron oxide nanobeads. These nanobeads facilitated rapid isolation of exosomes from the neutrophil supernatant, and they also enhanced the tumor-targeting capabilities of neutrophil-derived exosomes. Their findings indicated that neutrophil-derived exosomes exerted a notable anti-cancer effect by activating apoptosis signaling pathways. To further improve production efficiency, they created neutrophil exosome-like nanovesicles through a continuous extrusion process as a substitute for natural exosomes (**Figures 8**A and B). Under an external magnetic field, DOX-loaded superparamagnetic iron oxide nanoparticle-modified neutrophil exosome-like nanovesicles displayed biological functions similar to natural exosomes, selectively accumulating at tumor sites 189. An alternative strategy for producing exosome-like vesicles with an enhanced regenerative capacity at a high yield is through extrusion of neutrophils (**Figure 8**C). These exosomes-mimics and exosomes share a similar size range and the same surface protein composition. Sun et al. utilized this method to prepare exosome mimics from polymorphonuclear neutrophils and loaded them with VEGF for chronic diabetic wound treatment. A significant increase in the exosome yield was obtained, and the properties of natural neutrophils exosomes were retained (**Figure 8**D). To enhance exosome retention at the wound site, an extracellular matrix (ECM) hydrogel was used as a carrier for the exosome mimics to improve wound healing outcomes 190.

### 6.3 Neutrophil-like cell-based systems

There are challenges in membranes or exosomes extraction from primary neutrophils such as restricted access to primary neutrophils and a low extraction yield. Neutrophil-like cells could be an alternative to primary neutrophils. These cells can be easily generated from accessible sources, such as peripheral blood mononuclear cells or induced pluripotent stem cells 191-194. These cells can be cultured *in vitro* to yield a high viable cell density. Consistent and reproducible neutrophil-like cells can be produced for the extraction process for neutrophil membranes or exosomes. The extraction method from single population cells could become simple, and a high yield can be achieved from these neutrophil-like cells. Therefore, the extraction process can be streamlined and scaled up 195. The advantages of neutrophil-like cells including accessibility and abundance, along with genetic modifiability, and they become a reliable and promising source for developing neutrophil-mimicking drug delivery systems for the treatment of inflammatory diseases and infections.

**Figure 9:**
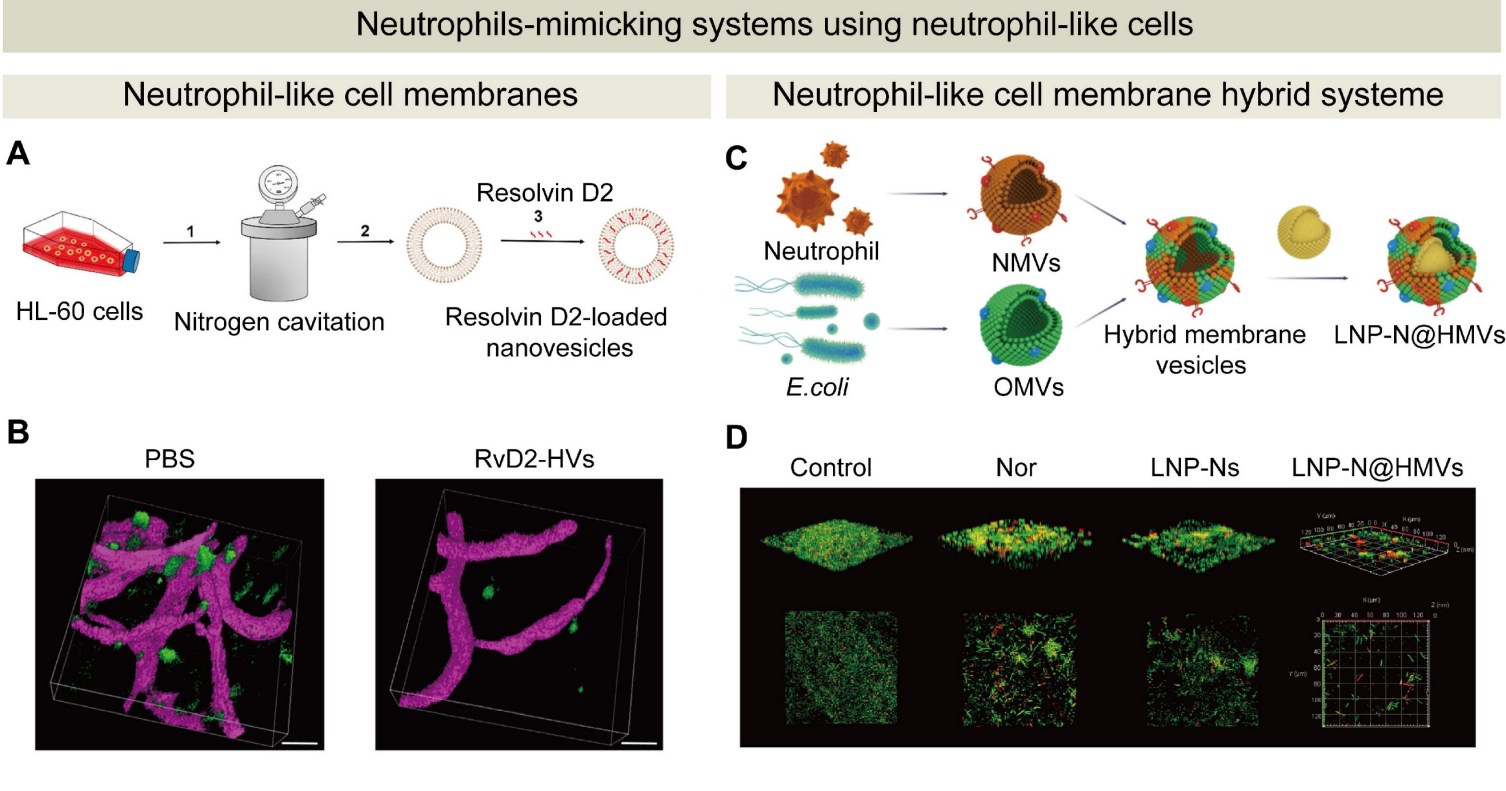
**Strategies of using neutrophil-like cells for targeted drug delivery.** (A) Design and fabrication of RvD2-loaded nanovesicles to target inflammatory brain endothelium during post-stroke reperfusion. (B) The RvD2-loaded nanovesicles reduced neutrophil infiltration in the middle cerebral artery occlusion mouse model, confirmed by intravital 3D microscopy. Scale bar: 20 μm. Adapted with permission from 197, copyright 2019 American Chemical Society. (C) Schematic illustration of the preparation process for LNP-N@HMVs, a hybrid membrane-coated antibiotic delivery system. (D) Confocal images of *E. coli* biofilms treated with various formulations at an equivalent norfloxacin concentration after live/dead staining. Adapted with permission from 199, copyright 2024 The American Association for the Advancement of Science.

The HL-60 cell line is commonly used to study neutrophil chemotaxis and their swarm behavior, because it can be differentiated to acquire functional characteristics of neutrophils. Irimia et al. demonstrated that dimethyl sulfoxide induced differentiated HL-60 cells exhibited a larger size and a greater length compared to primary neutrophils, thus these differentiated cells displayed a diminished capacity to navigate through narrow channels 196. Moreover, HL-60 is a cancer cell line, and its direct *in vivo* injection pose unpredictable risks. To address this, the cell membrane of neutrophil-like cells has been utilized to prepare neutrophil-mimicking drug delivery systems. Yang et al. reported a prodrug-loaded nanomedicine disguised with differentiated HL-60 cell membranes. The nanomedicine migrated to the ischemic brain tissue and released the drug locally in response to an elevated ROS level. Compared to intravenous administration, this nanomedicine delivered the drug at a dose 15.2 times higher than the nanomedicine without the cell membranes to the ischemic brain. Wang et al. developed HL-60 neutrophil membrane-derived nanovesicles loaded with Resolvin D2 for targeted delivery to inflammatory brain regions during reperfusion therapy (**Figure 9**A). Intravital 3D imaging revealed that the nanovesicles markedly reduced inflammation responses in post-ischemic brain tissues, observed as early as 2 h following administration after surgery (**Figure 9**B) 197. In another report, a neutrophil membrane-coated prodrug migrated to the ischemic brain tissue and release the loaded drug in situ in response to an elevated ROS level. Single-cell RNA sequencing analysis revealed that the prodrug reprogrammed microglial cells to an anti-inflammatory phenotype by regulating the activation of the NLRP3 inflammasome and the secretion of CXCL2 chemokine, thereby alleviating post-stroke inflammation 198. In addition to hybrid membranes formed from different cell type, a hybrid membrane could be created by combining neutrophil-like membranes with bacterial OMVs. Ding et al. leveraged the natural targeting properties of neutrophil membranes and the immunostimulatory effect of bacterial OMVs to create a neutrophil-mimicking drug delivery system (**Figure 9**C). This system not only enhanced the efficacy of antibiotics, but also reduced systemic toxicity, eventually effectively preventing bacterial infections (**Figure 9**D) 199. Notably, exosomes derived from neutrophil-like cells exhibited a comparable size range, a similar surface zeta potential, and equivalent protein compositions to those derived from primary neutrophils using conventional methods, and this production method achieved a 16-fold increase in the yield, suggesting neutrophil-like cells could be used as a practical alternative cell source to produce neutrophil-mimetic exosomes 200.

## 7. Emerging Approaches: Immunometabolism-Driven Development of NDDSs

Immunometabolism, the interplay between metabolic pathways and immune function, plays a pivotal role in neutrophil activation and fate decisions. Neutrophils exhibit marked metabolic plasticity, enabling rapid transitions from quiescence to activation to meet bioenergetic and biosynthetic demands 201-204. Circulating neutrophils primarily rely on cytoplasmic metabolic pathways—namely glycolysis and the pentose phosphate pathway (PPP)—as their principal energy sources to support phagocytosis, ROS production, and NET formation 205. However, under inflammatory conditions, mitochondrial metabolic processes—including fatty acid oxidation (FAO), the tricarboxylic acid (TCA) cycle, and oxidative phosphorylation—also play important roles in modulating neutrophil function in distinct activation states. Degranulation, the exocytic release of antimicrobial granule contents, is highly dependent on glycolysis-derived ATP production. Notably, hypoxia enhances neutrophil degranulation, contributing to epithelial injury 206. Chemotaxis is driven by orchestrated cytoskeletal remodeling, involving actin-rich pseudopod extension at the leading edge and myosin II-dependent uropod contraction at the rear, accompanied by cell polarization and adhesion. These processes are energy-intensive and tightly regulated by small GTPases. Mitochondria and TCA cycle enzymes have been shown to modulate chemotactic responses 207. For example, mitochondria at the leading edge release ATP to activate P2Y₂ purinergic receptors, amplifying chemotactic signaling via the mTOR pathway. Mitochondria in this region exhibit high membrane potential and calcium uptake capacity, enabling localized ATP release that sustains purinergic signaling cascades 208. Phagocytosis in neutrophils is predominantly fueled by glycolysis, with glucose serving as the primary energy substrate. Under conditions of extracellular glucose deprivation, neutrophils rely on glycogenolysis to mobilize intracellular glycogen stores and maintain phagocytic capacity 209. Activation is a prerequisite for NETosis, which is metabolically characterized by enhanced glycolysis, lipid metabolism, and oxidative burst. The PPP provides a substantial supply of NADPH, essential for NADPH oxidase-driven ROS generation. Meanwhile, glycolysis supports chromatin decondensation and nuclear envelope breakdown, both critical steps in the initiation of NET formation 210, 211.

Targeting neutrophil metabolic pathways offers a promising strategy to enhance drug delivery in inflammatory diseases. By modulating key metabolic circuits—such as glycolysis, fatty acid oxidation, or the pentose phosphate pathway—it may be possible to prolong neutrophil survival, enhance tissue infiltration, or augment effector functions like degranulation and NET formation. Such interventions could be leveraged to design metabolically guided neutrophil-based drug delivery systems that selectively release therapeutic agents at sites of inflammation or infection 212. This approach holds particular promise for precision targeting in hypoxic, immune-privileged, or otherwise inaccessible tissue microenvironments. Moreover, trained-immunity protocols have been developed to epigenetically and metabolically reprogram neutrophil phenotypes. The key characteristic of the trained immunity lies on epigenetic changes within innate immune cells after exposure to specific inflammatory signals. These epigenetic changes induce reprogramming of the metabolism of neutrophils, leading to enhancing their responses to the subsequent stimuli. Immune training of neutrophils and their progenitor cells can also yield a phenotype with therapeutic potential through adaptive selection. For instance, Bacillus Calmette-Guérin (BCG) vaccination has been shown to induce chromatin remodeling in neutrophils and enhance their ability to eliminate *Candida albicans* 213.

Moreover, it has been discovered that neutrophils displayed an anti-tumor effect after immune training by β-galactose. More specifically, β-glucan epigenetically reprogramed myeloid precursor cells and directed their differentiation towards neutrophils, particularly tumor-associated N1 neutrophils with the antitumor activity 214. It is worth noting that metabolic regulation may offer a novel approach to enhancing the antigen-presenting capacity of neutrophils, thereby contributing to anti-tumor immunity. A recent study by Wu et al. suggested that neutrophil-mediated antigen presentation may contribute to favorable prognosis across various cancer types. This function appeared to be modulated through leucine metabolism, which in turn promoted histone H3K27ac modifications that activated the antigen-presentation pathways. Moreover, these metabolically reprogrammed neutrophils were shown to elicit both neoantigen-specific and antigen-independent T cell responses 215. These findings suggest targeting neutrophil metabolic pathways, such as leucine metabolism, may enhance their immunostimmutory function in the TME, and metabolically reprogrammed neutrophils could be used for the development of immune-responsive NDDSs.

Metabolites of bacterial species within the human body can also reprogram neutrophil immunometabolism to modulate their phenotype, lifespan, and inflammatory responsiveness, which has a great impact on disease progression and therapeutic efficacy. Rewiring of the microbiome-driven metabolic pathway could accelerate aging of neutrophils and shift them into a pro-inflammatory state evidenced by enhanced activation and robust NET formation 216-218. For instance, certain bacterial species and their metabolites can modulate neutrophil infiltration, inflammatory signaling, and NET formation, thereby influencing tumor growth and metastasis. While some microbiome components suppress neutrophil-driven inflammation, others can promote tumor progression by inducing a shift into a pro-tumor neutrophil phenotype 219. Triner et al. reported that neutrophil depletion in combination with an increase in the number of intratumoral bacteria resulted in accelerated tumor growth, and exacerbated IL-17-dependent inflammation, indicating that neutrophils play a critical role in restraining tumor progression by diminishing bacteria-driven, IL-17-mediated inflammatory responses 220. By contrast, in a lung cancer model, the local microbiome triggered activated neutrophils to release cytokines, which subsequently promoted inflammation and tumor growth through lung-resident γδ T cells 221. Bacterial communities within the TME can also alter the behavior of neutrophils and other immune cells, influencing tumor progression 222. The interaction between neutrophils and various microbiome species in different disease context should be comprehensively examined and precise and personalized therapeutic strategies based on neutrophils could be developed. By incorporating epigenetic or metabolic modulators into the carriers, trained neutrophils can be delivered efficiently to target tissues, thus providing novel concepts and technical routes for the development of next-generation NDDSs.

## 8. Limitations and Challenges

Advances in single-cell technologies, imaging modalities, and high-throughput cytometric analyses have recently revealed neutrophil heterogeneity and functional plasticity in both human and animal models 41, 223-225. These insights have been rationally integrated into the development of NDDSs. By harnessing intrinsic chemotactic properties of neutrophils, their ability to traverse critical physiological barriers, and their immunomodulatory effects, neutrophils have been employed to prepare targeted drug delivery systems for a range of pathological conditions including inflammatory diseases, cancer, and infections. Innovative approaches, such as *ex vivo* drug loading, *in vivo* hijacking, surface modification, and genetic engineering, have been explored to enhance therapeutic outcomes of NDDSs. Meanwhile, neutrophil-derived membranes and exosomes, and neutrophil-like cells have been utilized to construct biomimetic drug delivery platforms. These biomimetic strategies surmount inherent limitations such as cell heterogeneity and short lifespans. More encouragingly, enhanced biocompatibility, efficient immune evasion, and reproducibility in therapeutic outcomes have been observed for neutrophil-derived biomimetic drug delivery systems in various animal models. These technologies have expanded the functional design of nanomaterials, paving the way for more versatile and targeted drug delivery systems in clinical applications.

**Figure 10:**
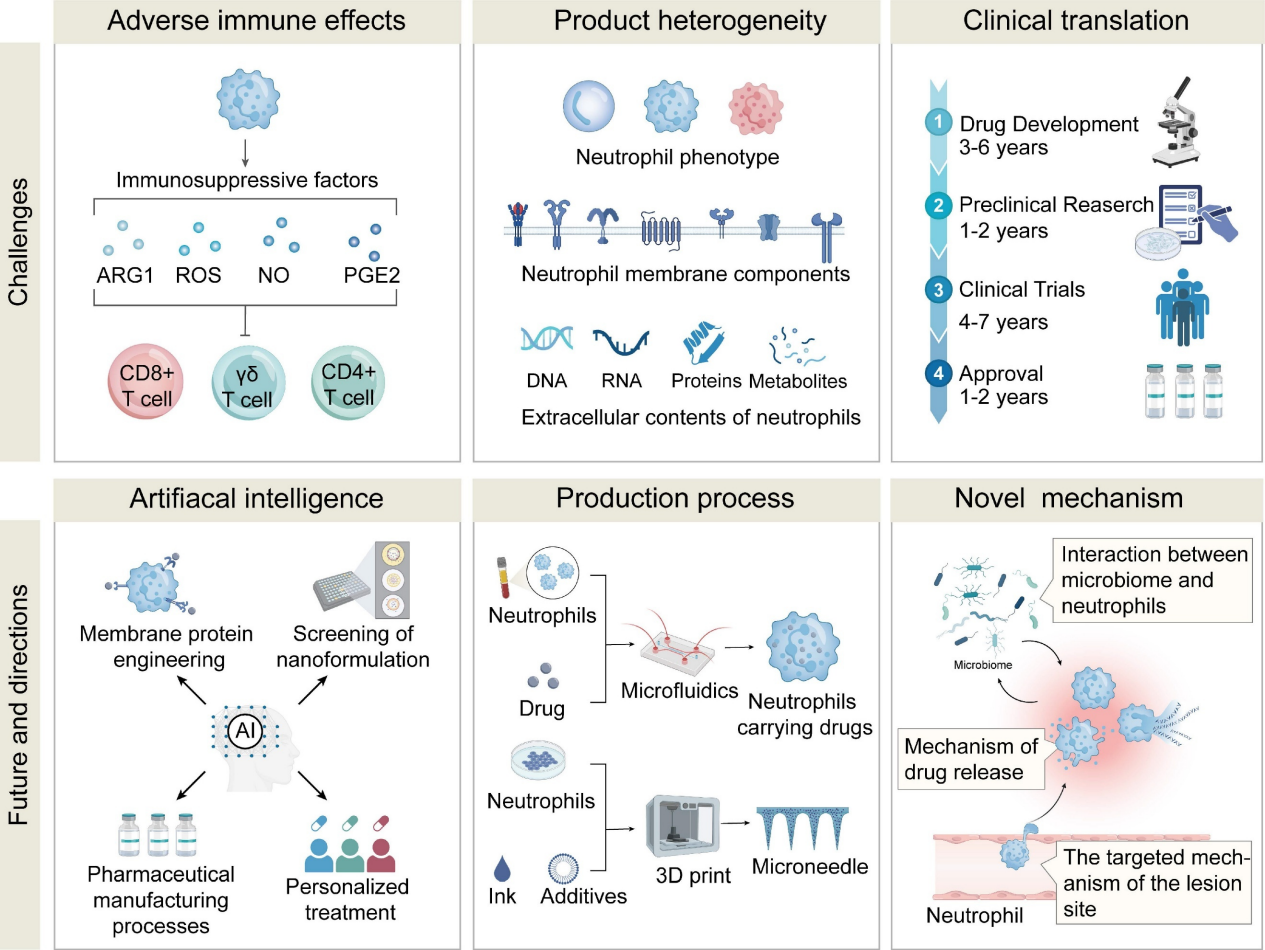
**Challenges and future perspectives of NDDSs.** NDDSs or neutrophil-derived biomimetic drug delivery systems face major challenges, including (1) unintended side effects after the activation of neutrophils; (2) product inconsistency due to the inherent heterogeneity of neutrophil-derived components; and (3) time-consuming and costly development of these drug delivery systems. To address these issues, future strategies can leverage artificial intelligence to optimize discovery and design of these drug delivery systems, employ microfluidic and 3D printing technologies to improve and standardize manufacturing processes, discover novel targeting mechanisms of neutrophils, control drug loading and release processes from NDDSs, and unveil the interplay between neutrophils and the microbiota.

Although a growing number of preclinical studies have demonstrated the potential of neutrophil-mediated drug delivery in targeting inflammatory diseases and tumors, very few of these preclinical studies have entered clinical trials and no registered clinical trials have been identified to specifically employ neutrophils as active drug carriers. Several fundamental challenges must be addressed before these systems can be successfully translated into clinical application (**Figure 10**): (1) the functional heterogeneity of neutrophils. Neutrophils exhibit context-dependent roles under different pathological conditions, and they could have pro-inflammatory and immunosuppressive effects. For example, in the TME, neutrophils can release immunosuppressive mediators such as arginase 1 (Arg1), ROS, nitric oxide (NO), and prostaglandin E2 (PGE2), which weaken antitumor immunity 41, 226-228. Neutrophils should be tuned to exhibit desirable therapeutic effects for specific disease contexts when they are employed for drug delivery systems; (2) product consistency. Neutrophils display marked differences in their physiological activities. These activities are associated with their maturation and activation states, which determines the surface protein composition and the biological functions. Moreover, the composition of cargos and membrane components in exosomes from neutrophils are varied from each batch, result in batch-to-batch product inconsistency for exosome-based drug delivery platforms 229-231; (3) clinical translation. Before clinical translation, neutrophil-based or neutrophil-derived biomimetic drug delivery systems must undergo comprehensive preclinical evaluation, with a particular focus on their safety. One major challenge lies in the complex immunological behavior of neutrophils, including their functional heterogeneity and dual roles in both promoting and relieving inflammation. Improper regulation may lead to excessive activation, tissue damage, or chronic inflammatory responses. Moreover, neutrophils interact dynamically with platelets, macrophages, and lymphocytes, potentially amplifying immune cascades and complicating therapeutic outcomes. These factors should be comprehensively addressed to modulate of the neutrophil activity within the drug delivery systems. Long-term safety assessment of NDDSs and neutrophil-derived mimetic drug delivery systems, especially on the risk of sustained inflammation post-treatment, is essential for their clinical translation. In addition, robust validation of the promising animal data in different disease-relevant models is critical for the translation of NDDSs to clinical settings. Scalable manufacturing processes should be developed to produce the product with reproducible critical quality attributes without significant batch variations with a GMP facility 232-234.

## 9. Conclusion and Future Perspectives

The integration of engineering, synthetic biology, and nanotechnology could overcome these hurdles of NDDSs for achieve precise targeting and controllable drug release at the targeted site (**Figure 10**). Artificial intelligence (AI) and machine learning algorithms can be used as powerful tools for high-throughput screening and rational design of NDDSs with unique characteristics including a high safety profile, a high level of biocompatibility, effective targeting moieties, a high drug loading rate, and controlled drug release kinetics. Additionally, AI-driven modeling of the neutrophil behavior in diverse pathological contexts can help formulating an optimal delivery route. For instance, AI has been applied to identify macropinocytosis as a key mechanism for enhancing lipid nanoparticle-mediated mRNA delivery, facilitating the optimization of intracellular drug transport in NDDSs 235. AI-driven analyses of the neutrophil behavior at different life stages have revealed their novel functions, including their immunological roles 236, 237, which could be harnessed for preparing novel NDDSs. In terms of delivery modalities, emerging technologies such as microfluidics and microneedle-based wearable systems could be explored to construct new NDDSs for spatially and temporally controlled administration of neutrophils. Microfluidic systems have been demonstrated to perform uniform membrane coating and cargo encapsulation and improve reproducibility and quality control of drug-loaded neutrophils-derived biomimetic systems 238, 239. Microneedle arrays offer localized and minimally invasive delivery, and they are particularly advantageous for treating cutaneous conditions, such as diabetic wounds, skin cancers, and hypertrophic scars 240-244. Integrating NDDSs with microneedles can improve the efficiencies in their tissue penetration and inflammatory site homing.

Moreover, elucidating fundamental mechanisms of neutrophil trafficking and drug release from neutrophils is essential for advancing this field. For instance, Wang et al. discovered a neutrophil-mediated mechanism to facilitate nanoparticle penetration into tumors by transiently disrupting the vascular basement membrane. In response to acute inflammation, activated platelets were rapidly recruited to accumulate at the perivascular niche in which there is a “nanoparticle pool”. These platelets subsequently promote the recruitment of neutrophils to the same site. As neutrophils transmigrated across the endothelium, they created transient basement membrane pores (approximately 2-6 μm in diameter) to trigger a burst-like release of nanoparticles, termed as “pool eruption”, into the tumor stroma. This mechanism significantly enhanced nanoparticle dispersion and penetration, and this is critically dependent on neutrophil extravasation and their capacity to remodel the basement membrane barrier 245. Although drug release from neutrophils via degranulation and NET formation has been observed, there are very few *in vivo* studies on controlled drug release from neutrophils.

In conclusion, we provided a comprehensive overview of the current progress of employing neutrophils or neutrophil components for drug delivery. We elucidated fundamental mechanisms of neutrophil targeting and drug release from neutrophils. Emerging strategies for harnessing these mechanisms for drug delivery were then elaborated, including both *in vivo* and *ex vivo* loading into neutrophils, preparation of biomimetic drug delivery systems from neutrophil membranes and exosomes. The latest advances in neutrophil-based drug delivery, such as neutrophil “drug backpacks”, CAR-neutrophils, and trained immunity, were captured to showcase their potential in treating cancer, inflammatory diseases and infections. Examples for these innovative strategies have consolidated that neutrophil-based platforms can confer novel therapeutic functionalities, while a precise, robust, and controllable method for delivering therapeutics in clinical setting via neutrophils or neutrophil components remains to be challenging. Nevertheless, advanced neutrophil-inspired drug delivery systems could be promising for treating diseases with difficult-to-overcome physiological barriers. These approaches not only offer valuable insights into the mechanisms of neutrophil-mediated micro/nanomaterials and their applications but also provide a strong foundation for the development of novel, highly effective drug delivery systems.

## Figures and Tables

**Table 1:** Engineering neutrophil-based strategies: modification methods and therapeutic mechanisms

Functional strategy	Therapeutic agent	Mechanism of Drug Loading/Targeting	Disease model	Treatment Modality	Ref.
*Ex vivo* drug loading	PTX-loaded liposomes carried by neutrophils	Extracellular co-culture	Post-surgical glioma	Neutrophils migrated to inflamed brain post-surgery and released PTX in situ to suppress residual tumor growth.	68
	Methotrexate-loaded liposomes uptaken by neutrophils	Extracellular co-culture	Myocardial ischemia/reperfusion injury	Neutrophils homed to inflamed sites and release drug-loaded liposomes, which were uptaken by macrophages to locally suppress inflammation and aid in tissue repair.	123
	Balloon contains ICG, miR-126a-5p	CD11b-Ab-surface modification /Extracellular co-culture	Atherosclerosis	Neutrophils guided the complex to plaques under shear stress, while ultrasound enhanced accumulation and enabled local release of anti-inflammatory agents for plaque stabilization.	133
	PTX- loaded liposomes, and an anti-PD-1-containing hollow ZGO@TiO₂ shell.	Extracellular co-culture	Glioblastoma	Neutrophils crossed the BBB and accumulated at tumor sites. Ultrasound activated TiO₂ to generate ROS, triggering drug release and immune activation	134
	DOX-loaded magnetic mesoporous silica NPs internalized by neutrophils.	Extracellular co-culture	Glioblastoma	Neutrophils loaded with magnetic mesoporous silica NPs were delivered to the tumor site, where they released DOX, reducing tumor recurrence. Magnetic resonance imaging tracked neutrophil migration.	25
	Poly(lactic-co-glycolic acid) nanoparticles encapsulating cabazitaxel or teriparatide	Extracellular co-culture	Mouse models of bone metastasis and osteoporosis	Leveraging the natural homing ability of senescent neutrophils to deliver drugs to the bone marrow, enhancing local drug concentrations and improving therapeutic efficacy	24
	DOX encapsulated in BSA NPs and delivered via neutrophils	Extracellular co-culture	Breast cancer model	LPS-induced inflammation helped recruiting NDDSs to tumors, promoted the release of NETs, and achieved local delivery of DOX	135
	Abraxane (albumin-bound PTX)/ Radiotherapy	Extracellular co-culture	Gastric cancer	Neutrophils naturally homed to inflamed tumor sites, where modest radiotherapy induced the release of inflammatory factors that triggered NET formation and burst release of Abraxane for enhanced tumor suppression.	136
	PLGA-based nanoparticle core coated with luteolin	Extracellular co-culture	Myocardial ischemia-reperfusion injury	Through neutrophil NET-mediated targeting of myocardial cells, luteolin was delivered to the sarcoplasmic reticulum, restoring calcium homeostasis and reducing oxidative stress.	129
Hitchhiking* in vivo*	Edaravone	Surface modification of liposomes with cRGD peptides	Cerebral ischemia	cRGD-modified liposomes (cRGDLs) crossed the BBB and delivered edaravone to ischemic brain regions with the help of monocytes and neutrophils as carriers	138
	LiMn₂O₄ nanozyme core	Surface modification of liposomes with cRGD peptides	Acute kidney injury	LiMn₂O₄ nanozyme mimicking SOD/CAT/GPx was loaded in cRGD-liposomes for neutrophil-mediated delivery; the enzyme-loaded liposomes targeted inflammation, scavenged ROS, and reduced oxidative stress and apoptosis	139
	GSK484-encapsulated ROS-responsive polymers	Surface modification with neutrophil-selective binding peptide	Traumatic brain Injuries (TBI) and stroke	NPs were delivered to the brain injury site via neutrophils hitchhiking, where ROS-triggered shell degradation induced the release of GSK484 to inhibit NETs and alleviated glia-mediated neuroinflammation.	140
	Baicalin encapsulated within tetrahedral framework nucleic acid	Surface modification with Ac-PGP peptide	Sepsis	The nanoplatform targeted neutrophils via Ac-PGP, facilitating baicalin delivery to inflammation sites and promoting M1-to-M2 macrophage polarization to alleviate inflammation and tissue damage.	141
	Ppa and Antibody (CD11b) functionalized gold nanorods	Conjugated with anti-CD11b antibodies	Carcinoma tumor	Photosensitization triggered neutrophil infiltration; NPs-CD11b were internalized by neutrophils and they were delivered to tumors	142
	Conjugation of IFN-β onto tannic acid nanoparticles.	Conjugated with anti-Ly6G antibody	Experimental Autoimmune Encephalomyelitis	Leveraging the anti-Ly6G targeting antibody and neutrophil-mediated NET release, IFN-β was specifically delivered to inflamed CNS sites, modulate immunity, reduced inflammation, and restored the motor function.	143
	Targeting peptide TP and sialic acid SA on a polydopamine coating of APTS	TP peptide targeting inflamed endothelial cells and SA targeting neutrophils	Neuroinflammation	The TP peptide targeted transglutaminase for neutrophil hijacking; SA enhanced uptake via L-selectin; and APTS reprogramed neutrophil death to apoptosis through ROS scavenging.	144
	OMVs coated on pioglitazone	OMVs serve as baits for neutrophil uptake.	Ischemic	Pathogen-mimicking nanoparticle crossed the BBB via neutrophils hijacking to release pioglitazone to reduce IL-1β by inhibiting the NLRP3 inflammasome.	145.
	Cisplatin encapsulated within a biodegradable NP core cloaked by bacteria-derived OMVs	OMVs serve as baits for neutrophil uptake	Solid tumor model	Pathogen-mimicking nanoparticles were internalized via recognition of pathogen-associated molecular patterns; they were released and engulfed by tumor cells upon PTT-induced local inflammation.	246
	Molecularly engineered liposomes	Rapid enrichment of iC3b through voluntary opsonization triggers neutrophil hijacking via CR3-mediated phagocytosis.	Lung inflammation	Phosphocholine liposomes bound to iC3b, triggering neutrophil uptake via CR3. Neutrophils delivered the drug to inflammation sites and released it through NETs or killing bacteria.	146
	PtCD nanozymes, piceatannol co-loaded into PLGA coated with platelet membranes	Platelet membrane proteins	Ulcerative colitis	Platelet membrane proteins on nanomaterials enhanced neutrophil phagocytosis. At the inflammation site, PtCD scavenged ROS to reduce oxidative damage, while Pic inhibited neutrophil adhesion.	147
	PLGA nanoparticles cloaked with a hybrid membrane of platelet-derived extracellular vesicles and calreticulin-expressed membrane	P-selectin targets activated neutrophils, homing to inflamed tissues via neutrophil migration	Acute Lung Injury, Severe Acute Pancreatitis	PC@PLGA targeted activated neutrophils via P-selectin and calreticulin acted as an “aged” signal, inducing macrophage-mediated premature macrophage-mediated programmed cell removal to relieve inflammation and prevent tissue damage.	148
Surface modification strategy	Liposomes loaded with a STING agonist	Maleimide (Mal) modification	TNBC	By leveraging the tumor-homing capability of activated neutrophils, STING agonists responded to hyaluronidase within tumors, thereby activating the STING pathway. This activation subsequently stimulated various immune cells, including dendritic cells, macrophages, and T cells, ultimately enhancing the efficacy of ICIs.	69
	CAMP and anti-CTLA-4 antibody combined therapy	Anti-CD11b Fab surface modification	Melanoma mouse model; 4T1 breast cancer mouse model	CAMP continuously activated neutrophils through integrin-mediated adhesion to form an anti-tumor N1 phenotype, thereby activating NK cells, DC cells and T cells and enhancing systemic anti-tumor immune responses.	150
	CAMP and anti-PD-1 antibody combined therapy	anti-CD11b Fab surface modification	Mouse GL261 model and brain GL261 model	Attaching micron-sized polymer patches (CAMPs) to neutrophils mechanically induced an anti-tumor phenotype, enhancing their ability to cross the BBB and activate T-cell immunity.	26
Genetic engineering	Chlorotoxin targeting CAR	Nucleofection	Glioblastoma	Engineered CAR neutrophils targeted GBM cells through CLTX-mediated binding (via MMP2 recognition), thereby enhancing cytotoxicity through direct cell contact and ROS generation to inhibit tumor growth.	153.
	CAR-neutrophils loaded with biodegradable mesoporous organic silica NPs	CRISPR-Cas9 and Extracellular co-culture	Glioblastoma	CAR-neutrophils efficiently traversed the BBB and homed to tumor sites where they internalized R-SiO₂ NPs; Drugs were released in a controlled, hypoxia-responsive manner, combining immune-mediated cytolysis with chemotherapeutic killing.	28

**Table 2:** Neutrophil-derived biomimetic delivery strategies: therapeutic components and mechanisms of action

Mimicking strategy	Components source	Therapeutic agent	Modifying methods	Disease model	Treatment modality	Ref.
Neutrophil membrane coating	Fresh human peripheral blood neutrophils	Polymeric core	Sonication	Arthritis	Mimicking natural neutrophil functions to neutralize pro-inflammatory cytokines, suppressing synovial inflammation, and protecting cartilage by acting as decoys for inflammatory mediators	161
	Peripheral neutrophils of mice	PLGA NPs loaded with Carfilzomib	Sonication	Breast cancer	The neutrophil-mimicking nanovehicle neutralized circulating tumor cells and disrupted metastatic niche formation	27.
	Mouse bone marrow	Polylactic acid NPs	Sonication	Liver inflammation	Neutrophil membrane-coated NPs mimicked neutrophil adhesion and homing to sequester inflammatory mediators, hindering inflammatory cell migration	162
	Neutrophils from mouse peripheral blood	PLGA NPs loaded with levofloxacin	Sonication	COPD with bacterial infection	The neutrophil membrane provided targeting and mucus penetration, allowing efficient levofloxaci delivery to reduce inflammation and bacterial infection	167
	Neutrophils from mouse peripheral blood	MOF containing Fe³⁺ nodes, loaded with porphyrin-NLS and PPE	Sonication	Glioblastoma	Camouflage with neutrophil membranes enabled tumor-specific targeting and BBB penetration. In the tumor environment, a high intracellular GSH level triggered MOF degradation to release porphyrin-NLS (for ¹O₂ generation under laser irradiation) and PPE, which synergistically induced histone H1 translocation and activated apoptosis pathways in cancer cells.	168
	Peripheral neutrophils of mice	Zeolitic imidazolate framework-8 (ZIF-8) loaded with anti-miR-155	Sonication	Atherosclerosis	Coating with neutrophil membranes enabled targeting via CD18-ICAM-1 interaction, while the ZIF-8 core provided a high drug loading and enabled efficient endosomal escape, effectively silencing miR-155 and reducing inflammatory mediators.	164
	Mouse bone marrow	PLA NPs	Sonication	Breast cancer	The NPs disrupted neutrophil-mediated tumor cell recruitment and cluster formation, significantly reducing metastatic, thereby preventing metastasis.	165
	Peripheral neutrophils of mice	Polymer nanoparticle core	Sonication and extrusion	Aortic dissection/aneurysm	Targeted delivery via neutrophils allowed controlled redox-triggered release of tRF-Gly-CCC to modulate inflammatory and smooth muscle cell pathways, reducing lesion progression and risks of vascular rupture.	166
Hybrid membrane coating	Peripheral neutrophil membranes and platelets membranes	DOX and ICG	Sonication	Breast cancer	Targeted elimination of CTCs and synergistic killing of tumors by photothermal therapy and chemotherapy	171
	RAW 264.7 cell membranes and mouse bone marrow neutrophil membranes	PLGA NPs loaded with RAPA	Sonication and extrusion	Glioma	Hybrid membrane-coated NPs penetrated through the BBB and achieved targeted glioma accumulation, while controllably released RAPA inhibited tumor growth and modulated the tumor immune microenvironment.	175
	Peripheral platelet membranes and Mouse bone marrow neutrophil membranes	Curcumin-loaded liposomes (CLs)	Sonication	Traumatic spinal cord injury	The biomimetic nanoplatform leveraged the targeting capacity of hybrid membranes to home to neuroinflammatory sites, degraded NETs via DNAse I, released curcumin in a controlled manner to modulate the NF-κB pathway, and facilitated neuronal repair.	172
	Fusion of neutrophil membranes and DOTAP (cationic lipids)	Mesoporous silica NPs (MSNs) loaded with miR-10b	Co-extrusion	Ischemic/reperfusion injury	The biomimetic nanoparticle targeted inflammatory myocardial tissues via neutrophil hijacking; it neutralized proinflammatory cytokines and promoted controlled miR-10b release to modulate the Hippo-YAP pathway and stimulated cardiomyocyte proliferation.	178
	Peripheral platelets membranes and Mouse bone marrow neutrophil membranes	BSA and PEI NPs loaded with R848	Sonication	Cancer surgery models	The biomimetic hybrid membrane targeted inflammatory wound sites and, aided by photothermal stimulation, R848 was released to reprogram macrophages and enhance T cell immunity, while CD47 blockade increased macrophage-mediated tumor cell clearance.	174
	RAW 264.7 cells and Mouse bone marrow neutrophils cell membrane	MnO₂-hydrangea nanoparticle core coated with PCN-224 MOF	Sonication	Orthopedic implant-associated biofilm infections in preclinical models	The NPN catalyzed H₂O₂ into oxygen, enhancing US-triggered SDT and disrupting bacterial homeostasis; concurrent release of Mn ions activated the cGAS-STING pathway, boosting immune responses	177
Neutrophil exosomes	Mouse bone marrow neutrophils- derived exosomes	DOX	Sonication	Glioma models	The neutrophil exosome-based system leveraged innate chemotactic properties to cross the BBB and target inflamed glioma, and releases DOX to kill tumor cells and improve survival.	187
	Mouse bone marrow neutrophils -derived exosomes	Prussian Blue NPs (uPB)	Copper-Free Click Chemistry	Rheumatoid arthritis	The hybrid platform targeted inflamed RA joints via neutrophil exosome proteins, scavenged ROS and neutralized cytokines, and rebalanced Th17/Treg cells to alleviate inflammation and protect cartilage.	188
	Peripheral blood neutrophils-derived exosomes	DOX decorated with superparamagnetic iron oxide nanoparticle	Extrusion	Tumor	The platform combined intrinsic apoptotic induction via neutrophil-derived exosomes with enhanced DOX delivery; superparamagnetic iron oxide nanoparticle decoration enabled magnetic targeting to tumor sites, thereby inhibiting tumor proliferation and extending survival.	189
	polymorphonuclear neutrophils from human peripheral blood	VEGF	Extrusion	Chronic diabetic wounds	The hybrid hydrogel combined bacteria-killing and cytokine-delivery with ECM-derived immune modulation to promote angiogenesis, regulate macrophage polarization, and enhance wound healing.	190
Neutrophil-like cells	HL-60	Self-assembly from ROS-responsive FTY720 monomers	Sonicattion	Ischemic stroke	Enhanced BBB penetration and targeted accumulation were realized via neutrophil membranes; ROS-triggered in situ FTY720 release promoted M2 microglial polarization and attenuated neuroinflammation	198
	HL-60	Resolvin D2 (RvD2)-	Sonication	Ischemic stroke	Targeted delivery to the inflamed brain endothelium was achieved via inherited adhesion molecules; in situ release of RvD2 attenuated inflammation and protected neural tissues	197
	HL-60 plasma membranes and microalgae	PLGA NPs	Sonication and then conjugation to microalgae using click chemistry	Acute Pseudomonas aeruginosa pneumonia	The microrobots actively navigated lung tissues using inherent algal motility, targeted deep lung regions via neutrophil membrane proteins, and released antibiotics to reduce bacterial burden with prolonged retention and minimal clearance.	163
	HL-60 plasma membranes with *E. coli*-derived OMVs	LNPs loaded with norfloxacin	Sonication	Systemic infection	Dual targeting through HMV enabled LNPs to accumulate at infection sites by binding to inflammatory endothelial cells and Gram-negative bacteria; controlled antibiotic release and immune activation, via OMVs, enhanced bacterial clearance and provided prophylactic protection	199

## Abbreviations

AI): Artificial intelligence
Bacillus Calmette-Guérin: BCG
blood-brain barrier: BBB
blood-brain tumor barrier: BBTB
C-X-C chemokine receptor type 4: CXCR4
catalase: CAT
central nervous system: CNS
chimeric antigen receptor: CAR
circulating tumor cells: CTCs
common myeloid progenitors: CMPs
complement receptor 3: CR3
chronic obstructive pulmonary disease: COPD
CpG: Cytosine-Guanine dinucleotide
Cyto-Adhesive Micro-Patches: CAMPs
doxorubicin: DOX
enhanced permeability and retention: EPR
extracellular matrix: ECM
Fc gamma receptors: FcγRs
fatty acid oxidation: FAO
glioblastoma: GBM
Granulocyte Colony-Stimulating Factor: G-CSF
granulocyte-monocyte progenitors: GMPs
hematopoietic stem and progenitor cells: HSPCs
hematopoietic stem cells: HSCs
Intercellular Adhesion Molecule-1: ICAM-1)
Intercellular Adhesion Molecule-2: ICAM-2
junctional adhesion molecules: JAMs
lipopolysaccharide: LPS
lymphocyte function-associated antigen 1: LFA-1
macrophage-1 antigen: Mac-1
maleimide: Mal
metal-organic framework: MOF
multipotent progenitors: MPPs
multiple sclerosis: MS
myocardial infarction: MI
nanoparticles: NPs
nature killer: NK
neutrophil-based drug delivery systems: NDDSs
neutrophil extracellular traps: NETs
neutrophil-derived exosomes: neutrophil-derived exosomes
nuclear localization signal-tagged porphyrin: porphyrin-NLS
outer membrane vesicles: OMVs
paclitaxel: PTX
pathogen-associated molecular patterns: PAMPs
platelet endothelial cell adhesion molecule-1: PECAM-1
poly(lactic acid): PLA
polyacrylamide: PAM
Poly(lactic-co-glycolic acid): PLGA
porcine pancreatic elastase: PPE
pentose phosphate pathway: PPP
P-selectin glycoprotein ligand-1: PSGL-1
Pyrophosphate-a: Ppa
reactive oxygen species: ROS
rheumatoid arthritis: RA
sialic acid: SA
superoxide dismutase: SOD
T-lymphocyte-associated protein 4: anti-CTLA-4
tricarboxylic acid: TCA
targeting peptide: TP
Toll-like receptors: TLRs
triple-negative breast cancer: TNBC
tumor microenvironment: TME
vascular cell adhesion molecule-1: VCAM-1
